# Super‐enhancer Activates Master Transcription Factor NR3C1 Expression and Promotes 5‐FU Resistance in Gastric Cancer

**DOI:** 10.1002/advs.202409050

**Published:** 2024-12-27

**Authors:** Junxian Yu, Mengdi Chen, Qingqing Sang, Fangyuan Li, Zhuoqing Xu, Beiqin Yu, Changyu He, Liping Su, Wentao Dai, Chao Yan, Zheng‐gang Zhu, Jiazeng Xia, Jianfang Li, Haoran Feng, Yunqin Chen, Yuan‐Yuan Li, Bingya Liu

**Affiliations:** ^1^ Department of General Surgery Shanghai Key Laboratory of Gastric Neoplasms Shanghai Institute of Digestive Surgery Ruijin Hospital Shanghai Jiao Tong University School of Medicine Shanghai 200025 China; ^2^ Department of Gastric Surgery Fujian Medical University Union Hospital Fuzhou 350001 China; ^3^ Shanghai‐MOST Key Laboratory of Health and Disease Genomics Shanghai Institute for Biomedical and Pharmaceutical Technologies Shanghai 200080 China; ^4^ Department of General Surgery Jiangnan University Medical Center Wuxi 200240 PR China

**Keywords:** 5‐Fluorouracil, gastric cancer, NR3C1, phase separations, super enhancers

## Abstract

Poor response to 5‐fluorouracil (5‐FU) remains an obstacle in the treatment of gastric cancer (GC). Super enhancers (SEs) are crucial for determining tumor cell survival under drug pressure. SE landscapes related to 5‐FU‐resistance are mapped to GC using chromatin immunoprecipitation‐sequencing (ChIP‐Seq). SiRNA transcription factors (TFs) screen determines master TF Nuclear Receptor Subfamily 3 Group C Member 1 (NR3C1) activated by SE. High NR3C1 expression driven by SE correlated with 5‐FU resistance in patient‐derived organoids (PDOs). Phase separation formed by NR3C1 is observed using fluorescence recovery after photobleaching (FRAP). NR3C1 protein and Mediator promoted SE‐related gene transcription via phase separation. SEs and NR3C1 co‐binding patterns are explored using Cleavage Under Targets and Tagmentation (CUT&Tag) sequencing. 5‐FU‐related genes driven by NR3C1 are identified using epigenetic reader inhibitor JQ1 and NR3C1 specific inhibitor Cort108297. NR3C1 knockdown increases 5‐FU sensitivity and alters the SE landscape through enhancer reprogramming, reducing downstream 5‐FU‐related target genes. JQ1 and Cort108297 both improve 5‐FU efficacy in PDOs and patient‐derived xenografts (PDXs) by destroying SEs or inhibiting NR3C1. In conclusion, SE‐driven NR3C1 promotes 5‐FU resistance in GC. SE destruction and NR3C1 inhibition lead to enhancer reconstruction and reduce 5‐FU‐related gene transcription, providing alternative therapeutic strategies for improving 5‐FU sensitivity.

## Graphical abstract

The patients with GC have poor response to 5‐FU. We identified that SE‐driven master TF NR3C1 increased 5‐FU resistance by forming phase separation droplets and promoting 5‐FU‐related gene transcription. NR3C1 knockdown changed SEs landscape through enhancer reprogramming. The epigenetic reader inhibitor JQ1 and NR3C1 specific inhibitor Cort108297 improved 5‐FU effectiveness in PDX models of GC.

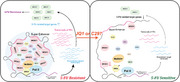

## 1 Introduction

The morbidity and mortality associated with gastric cancer (GC) remain high worldwide, especially in Middle and East Asian countries.^[^ ^1^ ^]^ Refractory GC is characterized by significant molecular heterogeneity, lack of effective therapeutic targets, and poor response to emerging targeted therapies and immunotherapies.^[^ ^2^, ^3^ ^]^ Chemotherapy remains a pivotal component in the comprehensive treatment of advanced GC. Fluorouracil (5‐FU) is a basic drug used in cancer chemotherapy with classic regimens such as FLOT (fluorouracil + oxaliplatin + docetaxel + leucovorin) and SOX (oxaliplatin + fluorouracil derivatives: S‐1).^[^ ^4^ ^]^ However, only some patients are sensitive to 5‐FU‐based chemotherapy.^[^ ^2^, ^3^ ^]^ Sensitivity to chemotherapy is determined by metabolic enzymes (such as thymidylate synthetase),^[^ ^5^ ^]^ transporters (such as ABC proteins),^[^ ^6^ ^]^ and other intrinsic characteristics.^[^ ^7^, ^8^ ^]^ Many attempts have been made to overcome this resistance, namely by targeting known factors such as the downregulation of multidrug resistance proteins (MDR1 and P‐gp),^[^ ^9^ ^]^ but none have proven effective. Exploring the mechanisms underlying 5‐FU resistance and developing effective interventions targeting these mechanisms may offer a promising alternative to solving drug resistance.

Super enhancers (SEs) are clusters of enhancers located in close proximity (full length <12.5 kb) that bind transcription factors and co‐regulators and intensively activate gene transcription.^[^ ^10^ ^]^ It is believed that the construction of active SE components and collaborating regulators, or SE landscape, is cell‐specific and spatial‐temporal specific, which is crucial for precise transcriptional regulation in cancer cells.^[^ ^10^, ^11^ ^]^ SEs can adapt within a certain range to regulate specific gene expression patterns at different spatial‐temporal levels, allowing cells to respond to different microenvironments, such as drug pressure.^[^ ^10^, ^11^ ^]^ It has been found that SEs can promote chemotherapy resistance in triple‐negative breast cancer, and when combined with bromodomain and extra‐terminal domain (BET) inhibitors, chemotherapy efficacy is enhanced, predominantly due to SEs disruption and transcription deregulation.^[^ ^12^ ^]^

Among SEs and SE‐related genes (SRGs), a cluster of SE‐driven transcription factors (TFs) can bind their own SEs, activate their self‐expression through positive feedback mechanisms, and form core transcription regulatory circuitries (CRCs).^[^ ^13^ ^]^ TFs in CRCs regulate numerous downstream target genes and play a central regulatory role in maintaining cellular identity and specific status.^[^ ^13^ ^]^ For instance, in limbal stem cells, a CRC composed of three master TFs is essential for maintaining corneal epithelial identity and homeostasis.^[^ ^14^ ^]^ Furthermore, it has been demonstrated that the master TF in osteosarcoma CRC promotes tumor growth and metastasis and induces sensitivity to chemotherapy.^[^ ^15^ ^]^ Although several studies have described the SE atlas of GC,^[^ ^16^ ^]^ most have focused on the role of SE in GC oncogenesis, while few studies have explored the impact of CRCs and master TFs on chemotherapy sensitivity in GC.

In this study, we characterized SEs and CRCs of 5‐FU sensitive and resistant GC cells. We found that high expression of the SE‐driven Nuclear Receptor Subfamily 3 Group C Member 1 (NR3C1) promoted 5‐FU resistance in GC cells and organoids. Inhibition of NR3C1 expression restored 5‐FU sensitivity. NR3C1 drove the formation of phase‐separation droplets around SEs and participated in the activation of several target genes related to 5‐FU resistance. In addition, we found that the epigenetic reader inhibitor JQ1 and the NR3C1 specific inhibitor Cort108297 both suppressed NR3C1 function by disrupting SEs or directly inhibiting NR3C1, thereby improving 5‐FU sensitivity in GC. These findings suggested a possible drug combination scheme for patients with GC undergoing 5‐FU‐basic chemotherapy.

## 2 Results

### 2.1 Characterization of SEs and CRCs Related to 5‐FU Resistance in GC

**Figure 1:**
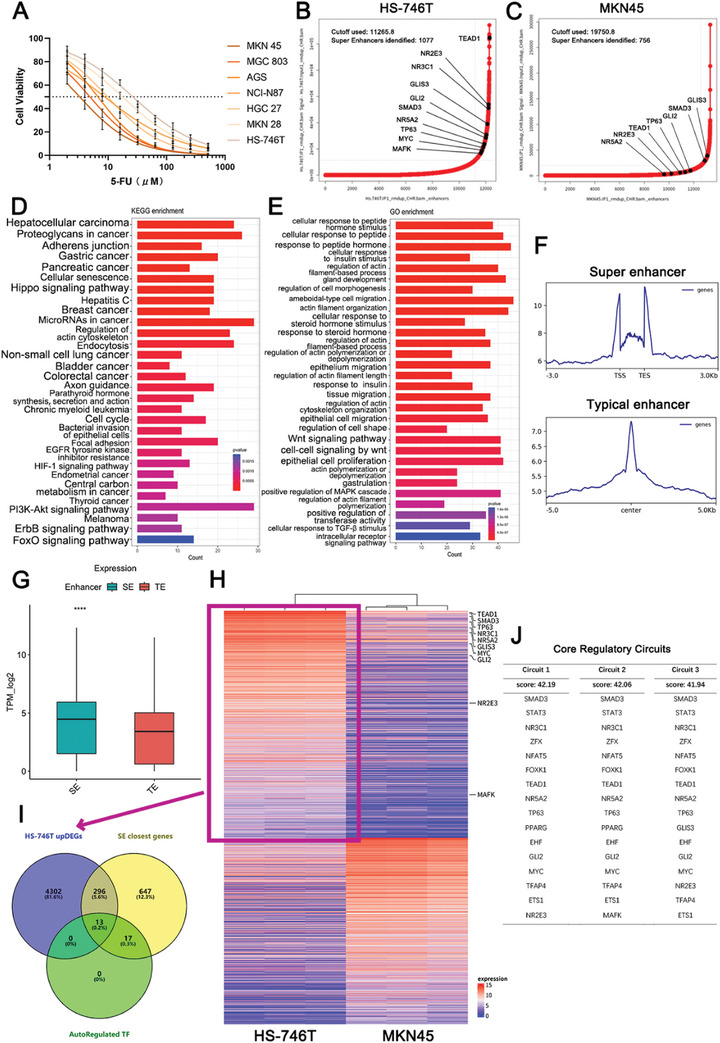
Super enhancer landscape and CRC of 5‐FU‐resistant GC cells. A) 5‐FU Sensitivity of common gastric cancer cell lines. B) Super enhancer (SE) curve of 5‐FU spontaneously resistant HS‐746T gastric cancer cells. C) SE curve of 5‐FU‐sensitive MKN45 gastric cancer cells. D) KEGG enrichment analysis of SE‐related genes (SRGs) in HS‐746T cells. E) GO enrichment analysis of SRGs in HS‐746T cells. F) Peak graphs of SE and Typical enhancers (TE) in HS‐746T. G) Transcriptional levels of SRGs and TE‐related genes (Student's *t*‐tests, two‐tailed). H) Differential expressed genes (DEGs) in HS‐746T and MKN45 cells. I) Venn map of SRGs, autoregulatory transcription factors (TFs), and HS‐746T high expression genes. J) Three CRCs of HS‐746T. NS: not significant, *p < 0.05, **p < 0.01, ***p < 0.001, ****p < 0.0001.

In seven GC cell lines, we found that MKN45 was the most sensitive to 5‐FU (IC_50_:3.274 µm), and HS‐746T was the most resistant to 5‐FU (IC_50_:26.50 µm), which was defined as primary 5‐FU resistance (**Figure** **1**A). Using chromatin immunoprecipitation sequencing (ChIP‐Seq) with an H3K27Ac antibody combined with the rank ordering of super‐enhancers (ROSE) algorithm,^[^ ^17^ ^]^ the SEs and SRGs of MKN45 (5‐FU‐sensitive) and HS‐746T (5‐FU‐resistant) cells were calculated and identified (Figure 1B,C). In HS‐746T cells, there were 1077 SEs identified with a threshold of 11 265.8 (Figure 1B). In MKN45 cells, there were 756 SEs identified with a threshold of 19 750.8 (Figure 1C).

As SEs influence cancer cells by directly activating the transcription of SRGs,^[^ ^10^ ^]^ pathway enrichment of SRGs may provide clues for understanding the biological processes hidden in 5‐FU resistant GC cells. Hence, SRGs in HS‐746T (5‐FU‐resistant; Figure 1D,E; Figure S1A, Supporting Information) and MKN45 cells (5‐FU‐sensitive, Figure S1B,C, Supporting Information) were analyzed using the Kyoto Encyclopedia of Genes and Genomes (KEGG) and Gene Ontology (GO) enrichment analyses, respectively. SRGs in both cell types were enriched in cancer‐related pathways, such as gastric cancer, colorectal cancer, and adherens junction. We further analyzed the effect of the SRGs on 5‐FU resistance in HS‐746T cells. SRGs were enriched in the cell cycle pathway related to 5‐FU resistance.^[^ ^18^ ^]^ The inhibition of cyclin‐dependent kinase 4/6 (CDK4/6) displayed a synergistic effect with 5‐FU in GC.^[^ ^19^ ^]^ In terms of cellular metabolism, SRGs were enriched in central carbon metabolism in cancer (CCM). The CCM is mainly involved in glycolysis, the pentose phosphate pathway, and the tricarboxylic acid cycle (TCA cycle).^[^ ^20^ ^]^ Abnormal CCM contributes to cancer stemness maintenance and redox imbalance,^[^ ^20^ ^]^ thereby enhancing resistance to chemotherapy drugs.^[^ ^21^ ^]^ Cellular signaling pathways, including the PI3K‐AKT, ErbB, and FoxO pathways, were enriched. The activation of the PI3K‐AKT pathway inhibits 5‐FU‐induced apoptosis and promotes 5‐FU resistance in GC, whereas the AKT pathway inhibition effectively restores 5‐FU sensitivity^[^ ^22^ ^]^ (Figure 1D). In the GO analysis, Wnt‐related signaling pathways were enriched (Figure 1E; Figure S1A, Supporting Information). These pathways are significantly correlated with the maintenance of cancer stemness and 5‐FU resistance in GC.^[^ ^23^ ^]^

Because pathway enrichment analysis cannot reflect gene transcription changes, we focused on SRGs’ transcription changes. The H3K27Ac peak values of SEs were significantly higher than those of typical enhancers (TE) (Figure 1F; Figure S1D, Supporting Information), which was consistent with previous reports.^[^ ^10^ ^]^ By combining ChIP‐Seq with RNA‐Seq, we found that the expression levels of SRGs were also significantly higher than those of the TE‐related genes (Figure 1G; Figure S1E, Supporting Information). The H3K27Ac peak distribution was mainly gathered around promoters (<3 kb), which supported the enhancer‐promoter interaction reported in other studies ^[^ ^24^ ^]^ (Figure S1F,G, Supporting Information).

There are nearly a thousand SEs and the range of SRGs is further extended. CRC, which is constructed from SE‐driven TFs, has been proposed to play a central regulatory role in maintaining a specific cellular status.^[^ ^13^ ^]^ Thus, we attempted to determine the specific CRC in HS‐746T (5‐FU‐resistant). Differentially expressed genes (DEGs) were identified between 5‐FU‐reluctant HS‐746T cells and 5‐FU‐sensitive MKN45 cells and highly expressed genes in HS‐746T cells (HS‐746T upDEGs, red box, Figure 1H) were used for subsequent analyses. Combining SRGs, HS‐746T upDEGs, and autoregulated TFs (based on prior knowledge of TF motifs), the CRCmapper algorithm calculated HS‐746T‐specific SE‐driven potential CRCs (https://bitbucket.org/young_computation/crcmapper/src/master/), and the three most probable CRCs were identified (the score in the table reflects the probability of each circuitry, Figure 1I,J). The determinants underlying 5‐FU resistance were narrowed down to the TFs listed in the CRCs.

### 2.2 Identification of Master TF NR3C1 that Correlated with 5‐FU Resistance

**Figure 2:**
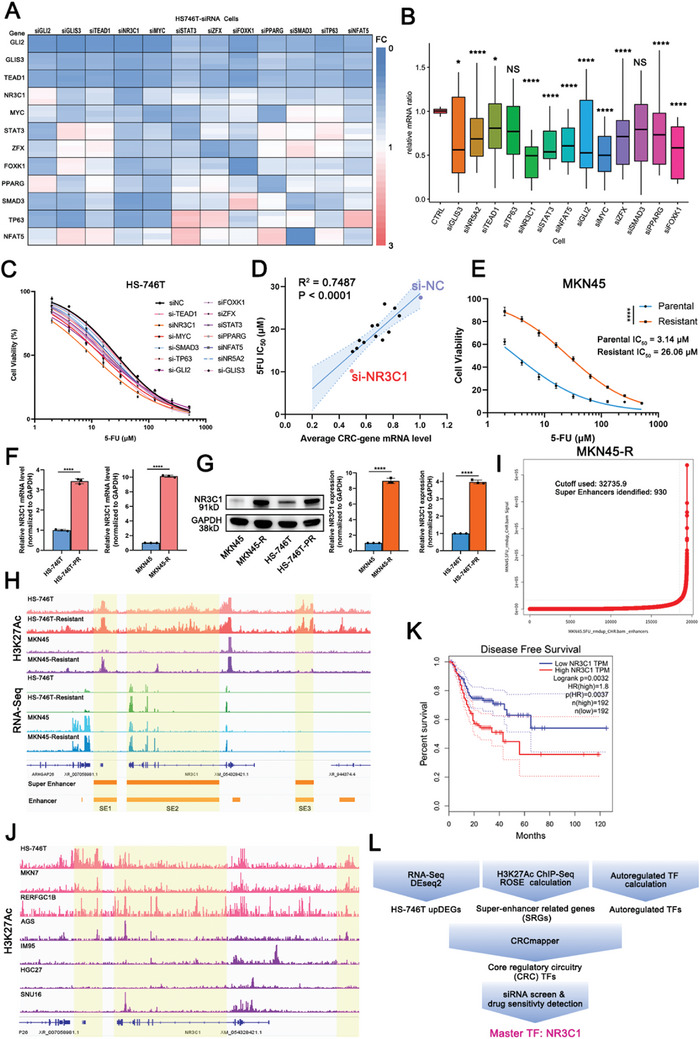
SE‐CRC‐driven NR3C1 correlated with 5‐FU resistance in GC cell. A) With each siRNA of CRC‐TF (*x*‐axis), the heatmap of each gene mRNA level (*y*‐axis: fold change of gene mRNA level to GAPDH) in CRC. B) With each siRNA of CRC‐TF, the transcription level of the whole CRC gene set (ANOVA, two‐tailed, mean ± SD). C) With each siRNA of CRC‐TF, the 5‐FU sensitivity of si‐TF gastric cancer cells. D) Correlation analysis between the transcription level of CRC and 5‐FU IC_50_ of gastric cancer cells (Spearman correlation test). E) IC_50_ of 5‐FU in MKN45‐R and parental cells (ANOVA, two‐tailed). F) The transcriptional levels of NR3C1 in MKN45 versus MKN45‐R, and HS‐746T versus HS‐746T‐PR cells (Student's *t*‐tests, two‐tailed, mean ± SD). G) The protein levels of NR3C1 in MKN45 versus MKN45‐R, and HS‐746T versus HS‐746T‐PR cells (Student's *t*‐tests, two‐tailed, mean ± SD). H) Around NR3C1, IGV map of H3K27Ac ChIP‐seq and RNA‐Seq of MKN45, MKN45‐R, HS‐746T, and HS‐746T‐PR cells. Yellow area showed the region of SE1, SE2, and SE3. I) SE curve of MKN45‐R cells. J) Around NR3C1, IGV map of 5‐FU sensitive and resistant gastric cancer cells in GSE186521. K) Disease‐free survival curves of the 384 patients in the gastric cancer cohort in GEPIA, with patients stratified based on NR3C1 expression levels (*n =* 384, Cox regression analysis). L) Flow chart of master TF NR3C1 identification from SRGs and CRC TFs. NS: not significant, *p < 0.05, **p < 0.01, ***p < 0.001, ****p < 0.0001.

To explore the contribution of each TF in CRC, small interfering RNA (siRNA) screening was performed on HS‐746T cells. The inhibitory effects of each siRNA on TF transcription in CRC (**Figure** **2**A), and on the whole CRC transcription (all TF mRNA levels in CRC as a set; Figure 2B) were assessed. The results showed that NR3C1 knockdown (siNR3C1) and MYC knockdown (siMYC) inhibited the entire CRC set to the greatest extent (Figure 2B). Simultaneously, the effects of each interference on 5‐FU sensitivity were examined, showing that siNR3C1 importantly improved the 5‐FU sensitivity of GC cells (siNR3C1:10.16 µm, *p <* 0.0001; siNC: 27.37 µm, Figure 2C). SiFOXK1, siGLI2, siMYC, and siSTAT3 displayed similar effects on 5‐FU sensitivity, whereas the knockdown of some TF did not affect 5‐FU sensitivity, such as TEAD1 (Figure 2C; Figure S2, Supporting Information). Correlation analysis between the expression levels of the CRC set and the 5‐FU IC_50_ value showed that the higher the expression of the whole set, the higher the 5‐FU IC_50_ value (R^2^ = 0.7487, *p <* 0.0001; Figure 2D). NR3C1 knockdown inhibited the entire CRC set the most significantly, making GC more sensitive to 5‐FU (Figure 2D). Therefore, we focused on NR3C1 to explore the molecular mechanisms underlying SE‐driven, TF‐mediated resistance to 5‐FU in GC.

5‐FU resistant cells, MKN45‐R, were screened and constructed by long‐term culture in 5‐FU medication, and IC_50_ value of MKN45‐R increased more than eight times (IC_50_ of Resistant vs Parental was 26.06 µm vs 3.14 µm, Figure 2E). 5‐FU partially resistant cells, HS‐746T‐PR, were also screened and constructed, and the IC_50_ value was increased by about two‐fold (Partly resistant: 58.97 µm, Parental: 26.29 µm, Figure S3A, Supporting Information). The mRNA and protein expression of NR3C1 were significantly increased in 5‐FU resistant cells (MKN45‐R and HS‐746T‐PR) compared with those of their parental cells (MKN45 and HS‐746T, Figure 2F,G). The SE landscapes of MKN45‐R and HS‐746T‐PR were described, and regions around NR3C1 were displayed in Integrative Genomics Viewer (IGV)^[^ ^25^ ^]^ graphs to determine the impact of long‐term medication on SE characteristics (Figure 2H,I; Figure S3B–D, Supporting Information). HS‐746T cells had three SE regions, SE1 (chr5: 143233723‐143262337), SE2 (chr5: 143275273‐143391380) and SE3 (chr5: 143486735‐143509814), near the NR3C1 gene. MKN45 cells had no enhancers in these regions, whereas MKN45‐R cells generated new enhancers in the SE1 and SE2 regions (top Figure 2H). HS‐746T‐PR cells also showed increased peak intensities in these regions (top Figure 2H). Additionally, RNA‐seq analysis revealed that NR3C1 expression levels in MKN45‐R and HS‐746T‐PR cells were higher than those in their parental cells (bottom Figure 2H).

For external validation, we combined 5‐FU sensitivity data from the Genomics of Drug Sensitivity in Cancer (GDSC) database^[^ ^26^ ^]^ with previous H3K27Ac ChIP‐Seq data of GC cells from Patrick Tan's study (GSE186521).^[^ ^27^ ^]^ Consistently, all 5‐FU resistant cells (top three cell lines: HS‐746T, MKN7, and RERFGC18) had SEs or enhancers around the SE regions of NR3C1, while only a few enhancers were present around the SE regions of NR3C1 in 5‐FU sensitive cells (bottom four cell lines: AGS, IM95, HGC27, and SNU16) (Figure 2J). Moreover, NR3C1 expression correlated with disease‐free survival (DFS) in patients with GC, with a hazard ratio (HR) of 1.8 (*p* = 0.0032) in Gene Expression Profiling Interactive Analysis (GEPIA) (http://gepia.cancer‐pku.cn/),^[^ ^28^ ^]^ indicating that patients with high NR3C1 expression had shorter DFS and worse prognosis (Figure 2K). The flow chart of master TF identification in GC is displayed in Figure 2L.

### 2.3 High NR3C1 Expression Promotes 5‐FU Resistance of GC Cells

**Figure 3:**
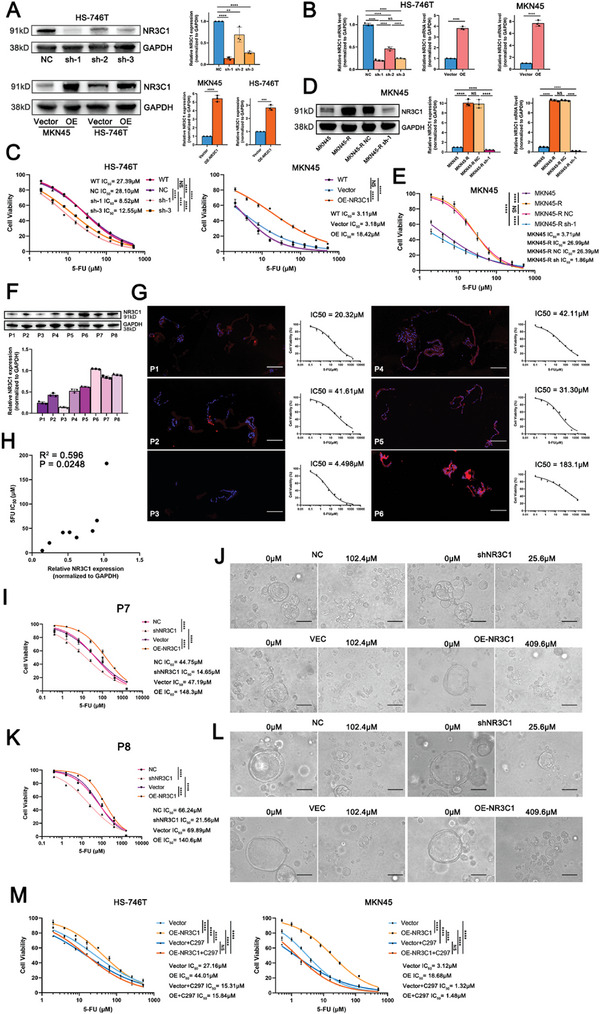
High NR3C1 expression promoted the 5‐FU resistance of GC cells. A) NR3C1 expression knockdown by shRNA in HS‐746T (top) and NR3C1 overexpression in HS‐746T and MKN45 (bottom), detected by Western blotting (Student's *t*‐tests, two‐tailed, mean ± SD). B) NR3C1 knockdown and overexpression in HS‐746T (left) and NR3C1 overexpression in MKN45 (right), detected by quantitative reverse transcription PCR (qRT‐PCR) (Student's *t*‐tests, two‐tailed, mean ± SD). C) IC_50_ values of 5‐FU in NR3C1 knockdown HS‐746T cells (left), and IC_50_ of 5‐FU in NR3C1 overexpressed MKN45 cells (right) (ANOVA, two‐tailed). D) NR3C1 knockdown detection in protein (left) and mRNA (right) levels in MKN45‐R (ANOVA, two‐tailed, mean ± SD). E) The 5‐FU IC_50_ values of MKN45, MKN45‐R, MKN45‐R NC, and MKN45‐R sh‐1 cells (ANOVA, two‐tailed). F) NR3C1 expression level of organoids cultured from gastric cancer tissues of patients 1–8 (P1‐P8). G) The 5‐FU IC_50_ values and NR3C1 immunofluorescence levels of organoids P1‐6. Scale: 100 µm. H) Correlation analysis of NR3C1 expression level and 5‐FU IC_50_ of gastric cancer organoids (Spearman correlation test). I) NR3C1 knockdown or overexpression in organoids P7, and 5‐FU IC_50_ values of indicated groups (ANOVA, two‐tailed). J) Photos around the IC_50_ values of the organoid P7 in each group. Scale: 100 µm. K) NR3C1 knockdown or overexpression in organoids P8, and 5‐FU IC_50_ values of indicated groups (ANOVA, two‐tailed). L) Photos around the IC_50_ values of the organoid P8 in each group. Scale: 100 µm. M) The 5‐FU IC_50_ values of HS‐746T, HS‐746T‐OE, MKN45, and MKN45‐OE with Cort108297 usage (ANOVA, two‐tailed). NS: not significant, *p < 0.05, **p < 0.01, ***p < 0.001, ****p < 0.0001.

With protein expression tested in seven gastric cancer cell lines (Figure S3E, Supporting Information), NR3C1 was knocked down using three shRNA lentiviruses in HS‐746T cells and overexpressed with the NR3C1 vector in MKN45 and HS‐746T cells (HS‐746T‐OE and MKN45‐OE cells) (**Figure** **3**A). The effect of shRNA2 was weak; therefore, shRNA1 and shRNA3 were selected for subsequent experiments (HS‐746T‐sh‐1 and HS‐746T‐sh‐3). In HS‐746T cells, NR3C1 knockdown significantly decreased the IC_50_ values (sh‐1:8.52 µm, sh‐3:12.55 µm, NC: 28.1 µm, *p <* 0.0001), and NR3C1 overexpression increased the IC_50_ values (OE: 42.12 µm, Vector: 26.87 µm, *p <* 0.0001; Figure 3B,C; Figure S4A, Supporting Information). In MKN45 cells, NR3C1 overexpression significantly increased the IC_50_ value (OE: 18.42 µm, Vector: 3.18 µm, *p <* 0.0001). Subsequently, shNR3C1 lentivirus was transinfected into 5‐FU resistant cells (MKN45‐R‐sh‐1, HS‐746T‐PR‐sh‐1), and found that NR3C1 knockdown effectively reduced IC_50_ values of 5‐FU resistant cells (MKN45‐R‐NC: 26.39 µm, MKN45‐R‐sh‐1:1.86 µm, *p <* 0.0001; HS‐746T‐PR‐NC: 59.38 µm, HS‐746T‐PR‐sh‐1:7.04 µm, *p <* 0.0001), restoring the 5‐FU sensitivity of resistant cells (Figure 3D,E; Figure S4B,C, Supporting Information).

NR3C1 expression and IC_50_ values were tested in eight patient‐derived organoids (PDOs) constructed from patients with GC (numbered P1‐P8) (Figure 3F,G, Figure S4D,E, Supporting Information), indicating that NR3C1 expression was positively correlated with 5‐FU IC_50_ values in the organoids (*R* ^2^ = 0.596, *p* = 0.0248, Figure 3H). Furthermore, NR3C1 expression was manipulated using lentivirus and control virus in the P7 and P8 organoids, respectively (P7‐sh, P7‐OE, P8‐sh, and P8‐OE). The IC_50_ values of NR3C1 knockdown PDOs were significantly decreased (P7‐sh: 14.65 µm, P7‐NC: 44.75 µm, *p <* 0.0001; P8‐sh: 21.56 µm, P8‐NC: 66.24 µm, *p <* 0.0001), while IC_50_ values of NR3C1 overexpressed PDOs were significantly increased (P7‐OE: 148.3 µm, P7‐Vector: 47.19 µm, *p <* 0.0001; P8‐OE: 140.6 µm, P8‐Vector: 69.89 µm, *p <* 0.0001) (Figure 3I–L; Figure S5, Supporting Information). These results indicated that NR3C1 knockdown improved 5‐FU sensitivity in GC organoids, and NR3C1 overexpression led to 5‐FU resistance. Cort108297 (C297) is a selective NR3C1 antagonist with no affinity for other steroid receptors.^[^ ^29^ ^]^ When C297 inhibited NR3C1 function, 5‐FU IC_50_ values in HS‐746T and MKN45 cells decreased significantly (Figure 3M).

### 2.4 NR3C1 Forms Droplets with SEs by Liquid‐Liquid Phase Separation (LLPS)

NR3C1 is a master TF regulated by SE in CRC, indicating that it binds to the promoters and enhancers of other SE‐associated TFs. Therefore, NR3C1 may be an important component of the central protein transcription machinery at transcription start sites (TSS) and SE sites. In the nucleus, the central transcription complex formed by TFs and co‐regulators condenses to form LLPS droplets, inside which SEs, target gene promoter regions and TFs are highly condensed, inducing intensive transcriptional tornados.^[^ ^30^, ^31^ ^]^

**Figure 4:**
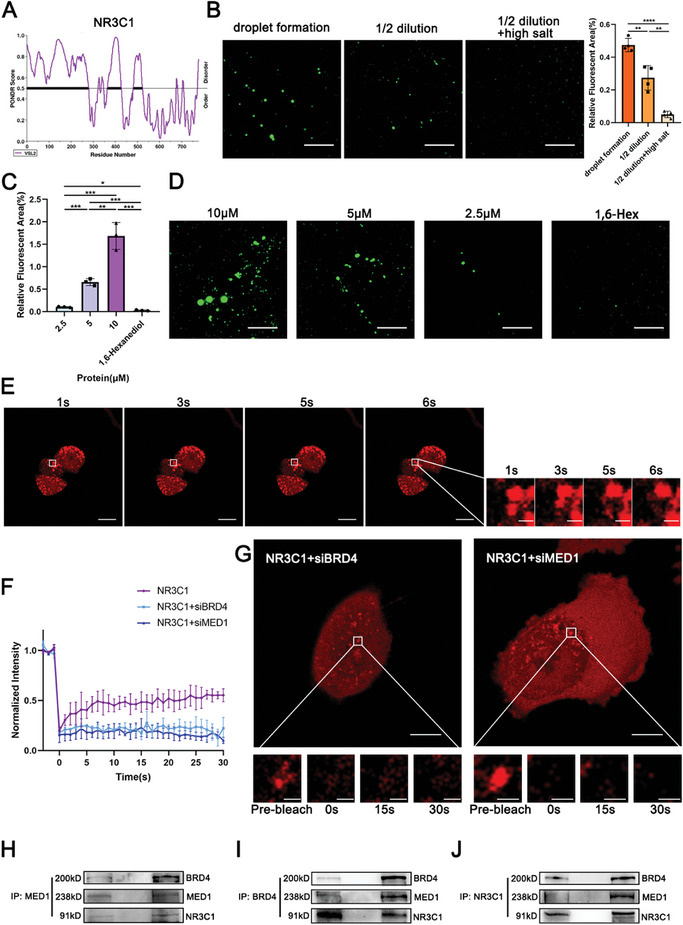
NR3C1 formed phase separation. A) Diagram of the protein disordered region (IDR) in NR3C1. B) The purified EGFP‐NR3C1 protein formed droplets, which were inhibited by reducing protein concentration and high salt solution (ANOVA, two‐tailed, mean ± SD). Scale: 5 µm. C) Statistical plots of the relative fluorescent area of droplets with different protein concentrations (ANOVA, two‐tailed, mean ± SD). D) Pictures of droplets with different protein concentrations and with the use of 1, 6‐hexadiol which destroys the droplets. Scale: 5 µm. E) The mCherry‐NR3C1 protein droplets in the nucleus of HS‐746T. Left: scale: 10 µm, right: scale: 1 µm. F) FRAP curves of mCherry‐NR3C1 in the nucleus of HS‐746T, and FRAP curves with si‐BRD4 and si‐MED1 respectively. G) Time series of FRAP images of mCherry‐NR3C1 protein with si‐BRD4 and si‐MED1. Top: scale: 10 µm, bottom: scale: 1 µm. H) Anti‐MED1 as a co‐immunoprecipitated antibody, detection of its binding to BRD4 and NR3C1. I) Anti‐BRD4 as a co‐immunoprecipitated antibody, detection of its binding to MED1 and NR3C1. J) Anti‐NR3C1 as a co‐immunoprecipitated antibody, detection of its binding to BRD4 and MED1. NS: not significant, *p < 0.05, **p < 0.01, ***p < 0.001, ****p <0.0001.

Intrinsically disordered region (IDR) is the physical substantial of LLPS droplet formation.^[^ ^31^ ^]^ We estimated the IDRs of CRC TFs using PONDR (http://www.pondr.com/)^[^ ^32^ ^]^ and found that some TFs, such as EHF, and PPARG in the CRC did not show IDRs, while other TFs, including NR3C1, had IDRs (**Figure** **4**A; Figure S6, Supporting Information). After the NR3C1 fluorescent protein was purified and smeared on slides, confocal microscopy revealed that the NR3C1 protein formed droplets in real‐time (Figure 4B). The quantity and size of the droplets are affected by the protein concentration, salt concentration, and the presence or absence of the phase‐change inhibitor 1,6‐hexadiol (1,6‐Hex) in the tubes.^[^ ^31^ ^]^ With double dilution of protein, droplets were reduced in number and size, and droplets were almost invisible with double protein dilution plus high salt concentrations (Figure 4B). As the protein concentration decreased, the number and size of droplets reduced, and droplet formation was rare with 1,6‐Hex addition (Figure 4C,D). As a control for 1,6‐hexadiol,^[^ ^33^, ^34^ ^]^ 2,5‐hexadiol did not interfere with droplet formation (Figure 4C,D; Figure S7A, Supporting Information). This phenomenon indicated that NR3C1 protein could form LLPS droplets in the tubes.

Inside the cells, specific proteins can form locally concentrated droplets at the SEs in the nucleus, which are dynamic and fluid.^[^ ^31^ ^]^ After NR3C1‐mCherry plasmids were transfected into the cells, NR3C1 formed locally concentrated red droplets in the nucleus that fused dynamically (Figure 4E; Figure S7B and Movies S1 and S2, Supporting Information). Fluorescence recovery after photobleaching (FRAP) experiments indicated that the NR3C1 droplets recovered successfully after being bleached (Figure 4F; Figure S7C, Supporting Information). DNA fluorescence in situ hybridization (FISH) combined with NR3C1 immunofluorescence showed that NR3C1 co‐localized with NR3C1 SEs (Figure S8, Supporting Information). This showed that NR3C1 formed locally concentrated droplets in the SEs. BRD4 and MED1 are the core components of the central transcription complex at the SEs and TSS. After BRD4 knockdown (siBRD4) and MED1 knockdown (siMED1), NR3C1 droplets did not recover, suggesting that droplet formation by NR3C1 was mediated by BRD4 and MED1 (Figure 4F,G). Co‐immunoprecipitation experiments revealed pairwise interactions among NR3C1, BRD4, and MED1 (Figure 4H–J). Moreover, both ChIP‐PCR and ChIP‐qPCR showed that NR3C1, BRD4, and MED1 had binding sites in the SE region of NR3C1 (Figure S9A,B, Supporting Information). Therefore, NR3C1 formed droplets and promoted LLPS in SEs with BRD4 and MED1.

### 2.5 NR3C1 Knockdown Induces Enhancer Remodeling in GC Cells

**Figure 5:**
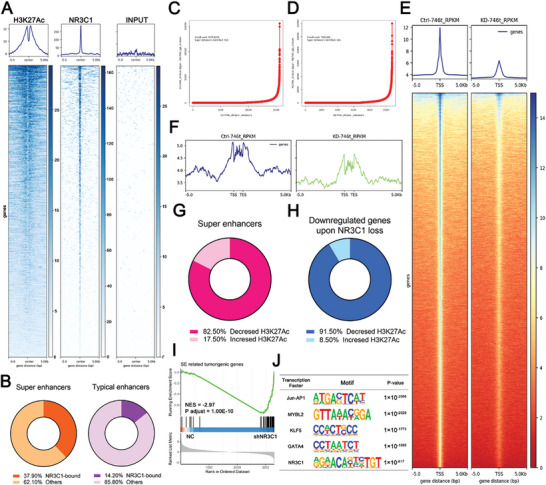
NR3C1 knockdown induced enhancer reconstruction of GC cells. A) Heatmaps of HS‐746T ChIP‐seq and NR3C1 CUT&Tag sequencing. B) The proportion of NR3C1 binding in SE and TE regions, respectively. C) and D) SE curves of the control group (left) and NR3C1 knockdown group (right). E) ChIP‐seq heatmaps in the control group (left) and NR3C1 knockdown group (right). F) Peak graphs of SE in the control group (left) and NR3C1 knockdown group (right). G) Pie chart showing the percentages of decreased or increased H3K27ac signals across the SEs of wild‐type cells upon NR3C1 loss. H) Pie chart showing the percentages of decreased and increased H3K27ac signals close to the loci of down‐regulated genes upon NR3C1 loss. I) GSEA of SE‐associated gene set in control versus shNR3C1 cells (Fisher's Exact test, one‐tailed). J) Motif de novo analysis of NR3C1 CUT&Tag sequencing (Hypergeometric test).

As a TF, the binding pattern of NR3C1 revealed by CUT&Tag‐seq indicated the potential function of NR3C1 to the downstream targets (**Figure** **5**A). In combination with H3K27Ac ChIP‐Seq, CUT&Tag‐seq showed that 37.9% of the SE region had NR3C1 binding sites, while only 14.2% of the TE region had NR3C1 binding sites, showing that the NR3C1 and SE regions had a higher overlap rate (Figure 5B). This indicated that NR3C1 was more likely to be highly enriched in the SE regions of GC cells.

Hence, we postulated that NR3C1 knockdown might change SE characteristics probably in GC cells, and described the SE landscape based on H3K27Ac ChIP‐Seq and RNA‐Seq (Figure 5C–E). The results showed that the control group had 913 SE regions (threshold: 9678.8223), whereas the number of SE regions was reduced to 686 in the NR3C1 knockdown group (threshold: 7902.489) (Figure 5C,D). The H3K27Ac peak values of SEs and TEs in NR3C1 knockdown cells both significantly decreased (Figure 5F; Figure S10A, Supporting Information), and 82.5% of the SEs have weakened H3K27Ac signals (Figure 5G). This suggested that NR3C1 knockdown significantly inhibited H3K27Ac signaling and changed the SE landscape of GC. RNA‐Seq analysis showed that 91.5% of the downregulated DEGs (NR3C1 knockdown vs NR3C1 control) had decreased H3K27Ac levels near the gene locus after NR3C1 knockdown (Figure 5H). Gene Set Enrichment Analysis (GSEA) revealed that the expression of the SRG set was significantly decreased in the NR3C1 knockdown group (NES = −2.97, p adjust = 1 × 10^−10^, Figure 5I), whereas the expression of the TE‐related gene set did not change (p adjust = 0.2, Figure S10B, Supporting Information). As demonstrated previously, NR3C1 participated in the transcription protein complex in SE regions through LLPS, indicating that NR3C1 bound to SEs to synergistically enhance downstream gene transcription and that NR3C1 knockdown could significantly inhibit the expression of SRGs by altering SE composition.

Multiple TFs usually bind to each other and form large protein complexes that act as transcription‐initiating machines at SEs, enhancers, and promoters.^[^ ^16^ ^]^ TFs inside the complex often cooperate to exert synergistic effects.^[^ ^16^ ^]^ In addition to the NR3C1 motif, the binding motifs of Jun‐AP1, MYBL2, KLF5, and GATA4 were found to be TF partners of NR3C1 using the hypergeometric optimization of motif enrichment (HOMER)^[^ ^35^ ^]^ algorithm (Jun‐AP1: p = 1 × 10^−2056^; MYBL2: p = 1 × 10^−2029^; KLF5: p = 1 × 10^−1773^; GATA4: p = 1 × 10^−1095^, Figure 5J). Studies have shown that Jun‐AP1 mediates 5‐FU resistance in GC by promoting the expression of nucleotide excision repair (NER) genes.^[^ ^36^ ^]^ In addition to accelerating the cell cycle and promoting GC cell proliferation,^[^ ^37^ ^]^ MYBL2 promotes the transcription of specific genes that mediate GC resistance to DNA‐damaged chemotherapy drugs.^[^ ^38^ ^]^ KLF5 has been shown to be an SE‐related core TF in a subtype of GC.^[^ ^39^ ^]^ KLF5 not only inhibits the apoptosis of GC cells,^[^ ^40^ ^]^ but also mediates GC resistance to DNA‐damaging chemotherapy drugs.^[^ ^41^ ^]^ GATA4 and KLF5 also cooperate to jointly maintain the oncogenic transcriptional regulatory network of GC and promote epithelial‐to‐mesenchymal transition (EMT) and GC metastasis.^[^ ^42^, ^43^ ^]^ Together, these results indicated that NR3C1 synergistically interacted with other TFs in common motifs and initiated downstream transcription to mediate 5‐FU resistance in GC cells.

### 2.6 Master TF NR3C1 Promotes 5‐FU Resistance Through Downstream Target Genes

**Figure 6:**
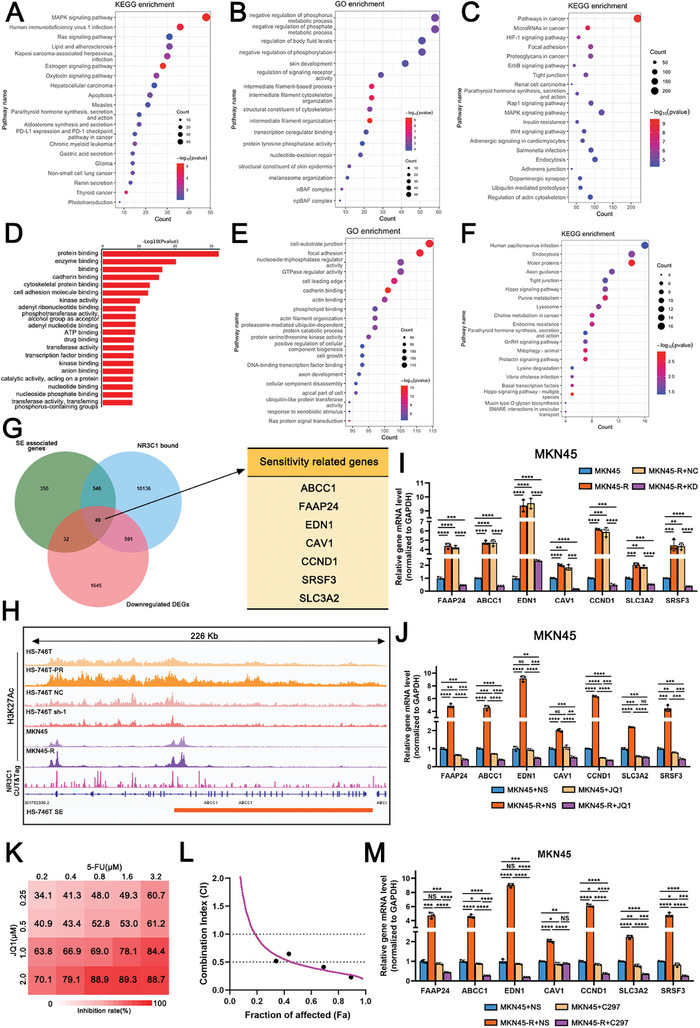
SE‐driven NR3C1 induces 5‐FU resistance in GC cells through activating downstream target gene clusters. A) KEGG enrichment analysis of SRGs in MKN45‐R. B) GO enrichment analysis of SRGs in MKN45‐R. C) KEGG enrichment analysis of NR3C1 binding genes based on CUT&Tag‐Seq. D) GO‐MF enrichment analysis of NR3C1 binding genes based on CUT&Tag‐Seq. E) Within RNA‐Seq of NR3C1 control group and knockdown group, GO enrichment analysis of differentially highly expressed genes in the control group. F) KEGG enrichment analysis of differentially highly expressed genes in the knockdown group in RNA‐Seq. G) Venn diagram of SRGs, NR3C1‐bound genes, differentially down‐regulated gene sets (left), and identification of a set of genes that are known to be relative to 5‐FU sensitivity (right). H) IGV map of H3K27Ac peak and NR3C1 CUT&Tag binding peaks around ABCC1 in HS‐746T, HS‐746T‐PR, HS‐746T NC, and HS‐746T‐sh‐1, MKN45 and MKN45‐R groups. I) The mRNA levels of 5‐FU‐related gene sets in MKN45, MKN45‐R, MKN45‐R NC, and MKN45‐R KD groups (ANOVA, two‐tailed, mean ± SD). J) The mRNA levels of 5‐FU‐related gene sets in MKN45+NS, MKN45‐R+NS, MKN45+JQ1, MKN45‐R+JQ1 groups (NS: normal saline, ANOVA, two‐tailed, mean ± SD). K) Synergistic effects of the combined use of JQ1 and 5‐FU in MKN45. Two drugs were double‐diluted (JQ1 in 4 gradients, 5‐FU in 5 gradients). The color scale for drug inhibition values. L) Fa (Fraction of affected)‐CI (Combination index) plots in MKN45 for the combination of JQ1 and 5‐FU. CI values of <0.7 indicate synergistic effects and CI values of <0.3 indicate strong synergistic effects. M) The mRNA levels of 5‐FU‐related gene sets in MKN45+NS, MKN45‐R+NS, MKN45+C297, MKN45‐R+C297 groups (ANOVA, two‐tailed, mean ± SD). NS: not significant, *p < 0.05, **p < 0.01, ***p < 0.001, ****p < 0.0001.

In 5‐FU resistant cells, based on the SE landscape and KEGG analysis, we found that SRGs were enriched in the MAPK and Ras signaling pathways, which maintained cell survival and proliferation. SRGs were also enriched in estrogen signaling pathways and gastric acid secretion, which are related to GC development (**Figure** **6**A; Figure S11A, Supporting Information). In GO enrichment analysis, SRGs were enriched in transcription co‐regulator binding, nucleotide‐excision repair pathways, regulation of signaling receptor activity, and protein tyrosine phosphatase activity (Figure 6B; Figure S11B, Supporting Information). These results suggested that SRGs activated a series of oncogenic signaling pathways, promoted cell repair mechanisms to recover from DNA drug‐induced damage, and maintained cell proliferation in 5‐FU resistant cells.

In addition to several oncogenic pathways, such as ErbB and Rap1 signaling pathways, NR3C1‐bound genes were enriched in the Wnt signaling pathway, which is strongly associated with cancer cell stemness and multidrug resistance (Figure 6C). Several pathways mediating the cell response to 5‐FU, including response to chemicals, cellular response to stimulus, kinase activity, drug binding, transcription factor binding, nucleotide binding, and nucleoside phosphate binding, were also enriched (Figure 6D). These results suggested that NR3C1 affected cell response to stimuli, such as external drugs, and activated multiple signaling pathways to maintain cell stemness and promote DNA drug resistance.

Moreover, DEGs (NR3C1 knockdown vs NR3C1 control) enriched in pathways like nucleoside‐triphosphatase regulator activity, DNA‐binding transcription factor binding, cell growth, and response to xenobiotic stimulus, were upregulated in the NR3C1 high‐expression group (control) (Figure 6E; Figure S11C, Supporting Information). DEGs enriched in the purine metabolism and the Hippo signaling pathway were upregulated in the NR3C1 low‐expression group (NR3C1 knockdown) (Figure 6F; Figure S11D, Supporting Information). These results suggested that NR3C1 responded to external stimuli and affected transcription regulation to maintain cell proliferation. Moreover, NR3C1 knockdown affected nucleotide metabolism.

Therefore, the intersection of SRGs, NR3C1‐binding gene sets, and down‐DEGs of the NR3C1 knockdown group were obtained to identify the direct target genes regulated by SE‐NR3C1 and related to drug resistance (Figure 6G). Among the 49 genes at this intersection, seven genes were previously reported to be correlated with 5‐FU‐related chemotherapy resistance, including FAAP24, ABCC1, EDN1, CAV1, CCND1, SRSF3, and SLC3A2. The multiple sequencing data in the IGV graphs indicated that NR3C1 bound to the SE regions of these genes and that the SE peaks were stronger in 5‐FU resistant cells than in parental cells (Figure 6H; Figure S12A–F, Supporting Information). Taking ABCC1 as an example, near the SE regions of ABCC1, NR3C1 had several binding sites. Furthermore, the peak intensity of ABCC1‐SE in HS‐746T‐PR was higher than that in HS‐746T, whereas that in HS‐746T‐sh‐1 was lower than that in the control group. The peak intensity of ABCC1‐SE was consistently higher in MKN45‐R cells than in MKN45 cells (Figure 6H). Both ChIP‐PCR and ChIP‐qPCR showed that NR3C1, BRD4, and MED1 had binding sites in the SE region of ABCC1, which were markedly inhibited by the BRD4 inhibitor, JQ1 (Figure S9A,B, Supporting Information). Moreover, dual‐luciferase assays indicated that NR3C1 bound to the ABCC1 SE region and ABCC1 SE promoted luciferase transcription as an enhancer (Figure S9C, Supporting Information). The transcription levels of the seven 5‐FU‐related genes in the 5‐FU resistant cells (MKN45‐R) were higher than those in the parental cells (MKN45). Their transcription was inhibited when NR3C1 was knocked down (MKN45‐R‐sh‐1), indicating that NR3C1 knockdown led to a loss of its promotional effect on 5‐FU‐related genes (Figure 6I).

FAAP24^[^ ^44^, ^45^ ^]^ and SRSF3^[^ ^46^, ^47^ ^]^ have been reported to participate in homologous recombination repair and promote 5‐FU resistance in cancer cells, in addition to the multidrug resistance protein ABCC1.^[^ ^48^ ^]^ In addition, downstream target genes promote 5‐FU resistance through ferroptosis via SLC3A2 and through the activation of Wnt signaling via CAV1.^[^ ^49^, ^50^ ^]^ Overexpression of CCND1 enhanced 5‐FU resistance in GC.^[^ ^51^, ^52^ ^]^ The results described above (Figure 6G–I) indicated that receiving drug signals, NR3C1 activated downstream target genes related to DNA repair to eliminate abnormal DNA produced by the 5‐FU attack, maintained cell homeostasis, and prevented cell death.

The epigenetic reader inhibitor JQ1 is a Bromo‐ and extra‐terminal domain family inhibitor (BETi), that inhibits BRD4 binding to chromatin.^[^ ^53^ ^]^ JQ1 causes SE destruction, increases apoptosis, and hampers SE‐induced oncogene transcription.^[^ ^53^ ^]^ ChIP‐PCR and ChIP‐qPCR showed that JQ1 significantly inhibited NR3C1, BRD4, and MED1 binding to both the NR3C1 SE and ABCC1 SE regions (Figure S9A,B, Supporting Information). In MKN45 cells, the results showed that JQ1 had a significant inhibitory effect on the transcription of five 5‐FU‐related genes, except for EDN1 and CAV1 (Figure 6J). MKN45‐R cells were treated with JQ1 and the results showed that JQ1 significantly inhibited all seven 5‐FU‐related genes (Figure 6J). The inhibitory effect of JQ1 on HS‐746T and HS‐746T‐PR suggested that drug‐resistant cells had a higher level of transcriptional addiction and were more dependent on SE‐driven transcriptional activation. Therefore, JQ1 exerted a more significant inhibitory effect on HS‐746T‐PR than on HS‐746T (Figure S13A,B, Supporting Information). Furthermore, the combination of JQ1 and 5‐FU exerted a synergistic effect under unfixed drug proportions in MKN45 cells (Figure 6K). The combination index (CI) of the two drugs under fixed drugs proportion (JQ1:5‐FU = 1.25:1) showed that JQ1 and 5‐FU had a synergistic effect with the CI <0.7 (Figure 6L). Similar findings were observed in HS‐746T with a CI <0.5 (Figure S13C,D, Supporting Information). The NR3C1 inhibitor C297 had an inhibitory effect on five 5‐FU‐related genes (WT+NS group vs WT+C297 group), except for FAAP24 and EDN1 (Figure 6M). In 5‐FU‐resistant cells, the transcription of all seven 5‐FU‐related genes was significantly inhibited by C297 treatment (Figure 6M; Figure S13E, Supporting Information). Together, these results indicated that both JQ1 and C297 could inhibit NR3C1 downstream 5‐FU‐related genes, especially in 5‐FU‐resistant cells.

### 2.7 SE Destruction and NR3C1 Inhibition Improve 5‐FU Sensitivity In Vivo

**Figure 7:**
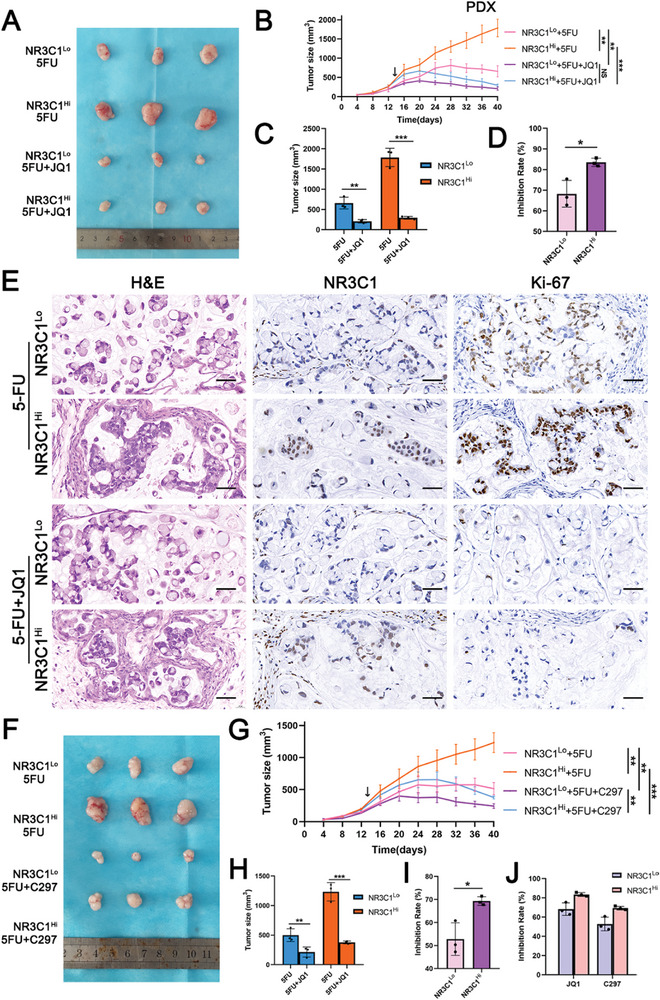
JQ1 and C297 reverses 5‐FU resistance in PDX models of GC. A) Images of NR3C1^Hi^ and NR3C1^Lo^ PDX tumors in 5‐FU group and 5‐FU+JQ1 groups (*n =* 3). B) Tumor growth curve of PDX tumors (ANOVA, two‐tailed). C) Tumor volume of each group at endpoint (Student's *t*‐tests, two‐tailed, mean ± SD). D) Inhibition rate of 5‐FU+JQ1 in each group at endpoint (Student's *t*‐tests, two‐tailed, mean ± SD). E) H&E, KI‐67 and NR3C1 staining of NR3C1^Hi^ and NR3C1^Lo^ PDX tumors. Scale bars: 50 µm. F) Images of NR3C1^Hi^ and NR3C1^Lo^ PDX tumors in 5‐FU and 5‐FU+C297 groups (*n =* 3). G) Tumor growth curve of PDX tumors (ANOVA, two‐tailed). H) Tumor volume of each group at endpoint (Student's *t*‐tests, two‐tailed, mean ± SD). I) Inhibition rate of 5‐FU+JQ1 in each group at endpoint (Student's *t*‐tests, two‐tailed, mean ± SD). J) Inhibition rate of 5‐FU+JQ1 and 5‐FU+C297 in each group. NS: not significant, *p < 0.05, **p < 0.01, ***p < 0.001, ****p < 0.0001.

Based on the findings in vitro, two GC patient‐derived xenograft (PDX) models were constructed and treated with the drugs (5‐FU, 5‐FU+JQ1, and 5‐FU+C297) (**Figure** **7**A–I). Drugs were administered for five consecutive days as a course of treatment and rested for two days before the next course. The animals were euthanized on the 40th day. The results showed that both 5‐FU+JQ1 and 5‐FU+C297 significantly inhibited tumor growth, leading to smaller tumors and slower tumor growth (Figure 7A–C,F–H), and fewer Ki‐67 positive cells compared with 5‐FU alone (Figure 7E; Figure S14, Supporting Information). Moreover, the combination of 5‐FU+JQ1 and 5‐FU+C297 had a higher inhibitory effect on NR3C1 high‐expression (NR3C1^Hi^) PDXs than on NR3C1 low‐expression (NR3C1^Lo^) PDXs (Figure 7D,E,I). These findings suggested that, in preclinical trials, NR3C1^Hi^ tumors benefit more from 5‐FU+JQ1 and 5‐FU+C297 than NR3C1^Lo^ tumors. For both drug combinations, a slight difference was observed in the inhibition rates of 5‐FU+C297 and 5‐FU+JQ1 (Figure 7J). These findings highlighted the clinical translational value of 5‐FU+JQ1 and 5‐FU+C297 as potential drug combinations for the treatment of 5‐FU‐resistant tumors.

## 3 Discussion

SEs have intrinsic cellular properties with lineage specificity, and many efforts have been made to explore their roles in maintaining cell identity^[^ ^31^, ^54^ ^]^ and in tumor development.^[^ ^55^, ^56^, ^57^ ^]^ Studies on GC have focused on revealing SE characteristics in different subtypes^[^ ^16^, ^27^ ^]^ and analyzing the molecular mechanisms by which SEs promote cell proliferation and metastasis.^[^ ^27^, ^39^ ^]^ Recent studies have shown SE‐mediated resistance to platinum and molecular inhibitors in lung and ovarian cancers.^[^ ^58^, ^59^ ^]^ As a chemotherapeutic drug, 5‐FU still has an unsatisfactory response in the treatment of patients with advanced GC. Few studies have elaborated on the role of SEs in 5‐FU resistance in GC. This study described the SE landscape and CRC involving SE‐driven TFs in 5‐FU‐reluctant GC cells. NR3C1 played a prominent role in transcriptional regulation and NR3C1 knockdown improved 5‐FU sensitivity. This study also found that new enhancers emerged in 5‐FU resistant cells to activate NR3C1 transcription, suggesting that GC cells promoted NR3C1 transcription through inherent SEs or through the emergence of new SEs to resist 5‐FU (Figure 2).

SEs and their target genes can be brought together through the flexible rotation of chromatin without a physical distance limit.^[^ ^30^, ^31^, ^32^ ^]^ This spatial DNA structure is present in protein LLPS droplets, which contain many concentrated TFs, the transcriptional central controller‐Mediator complex, RNA polymerase II (RNA pol II), and the important transcription regulatory factor BRD4. BRD4 and TFs recognize specific DNA regions, the Mediator complex and RNA pol II initiate transcription, and TFs further promote transcription.^[^ ^30^, ^31^ ^]^ The NR3C1 protein was observed to form dynamic droplets in tubes and nuclei (Figure 4), consistent with previous reports that the DNA‐binding domain of NR3C1 directed the selective partitioning of co‐regulators and protein condensation.^[^ ^60^ ^]^ Moreover, we found that NR3C1 bound to BRD4 and MED1 and that NR3C1 droplet formation was inhibited by siBRD4 and siMED1 (Figure 4), suggesting an important role for the NR3C1‐BRD4‐MED1 complex integrity in LLPS of the NR3C1 protein. Furthermore, the NR3C1 binding region significantly overlapped with more SEs than TEs, and NR3C1 knockdown significantly reduced the SE composition of cells and inhibited the overall transcription of SRGs (Figure 5), indicating that NR3C1 participated in SE composition and maintenance via NR3C1‐BRD4‐MED1 droplets.

The *NR3C1* gene encodes the glucocorticoid receptor that binds to glucocorticoid response elements. NR3C1 can activate gene transcription in a hormone‐independent manner.^[^ ^61^ ^]^ Dexamethasone and its analogs are commonly used to alleviate inflammation and adverse reactions to 5‐FU and other chemotherapeutic drugs for GC and other cancers. In addition to inhibiting the immune‐inflammatory response,^[^ ^61^ ^]^ NR3C1 mediates resistance to platinum and small‐molecule drugs in lung and ovarian cancers.^[^ ^62^, ^63^ ^]^ Recently, it was reported that glucocorticoids bound to NR3C1 and promoted neutrophil extracellular traps and cancer metastasis.^[^ ^64^ ^]^ In GC, we found that SE‐driven NR3C1 served as the master TF promoting 5‐FU resistance, bringing out the caution of dexamethasone application in patients with GC undergoing chemotherapy.

The BRD4 inhibitor JQ1 is a broad‐spectrum epigenetic modifier that obstructs transcription complex formation in SEs, and it has been reported that JQ1 can re‐sensitize drug‐resistant patients.^[^ ^65^, ^66^, ^67^ ^]^ This study preliminarily demonstrated the phenomenon of JQ1 sensitizing 5‐FU resistant cells. The clinical safety of combining the broad‐spectrum inhibitor JQ1 with chemotherapeutic drugs needs to be tested in future clinical trials. As reported in this study, the NR3C1 specific inhibitor Cort108297 also re‐sensitized 5‐FU resistant cells and PDXs, and it may be an alternative for the 5‐FU combination, requiring further exploration. The oriented release of NR3C1 inhibitor inside the tumor may also solve concerns regarding tumor chemoresistance caused by steroid administration. Multiple clinical trials on BRD4 inhibitors and NR3C1‐specific inhibitors have been carried out (such as NCT04309968: SYHA1801 in advanced solid tumors, NCT03205176: AZD5153 in refractory solid tumors, and NCT03776812: relacorilant in recurrent platinum‐resistant ovarian, fallopian tube, or primary peritoneal cancer), with drug efficacy results eagerly awaited. BRD4 inhibitors and NR3C1‐specific inhibitors require further clinical trials to test their efficacy in GC.

This study aimed to elucidate the mechanism of the key TF NR3C1 but fell short of delving deeper into the molecular mechanism of core regulatory circuitry (CRC) underlying 5‐FU resistance. In this study, NR3C1 was depleted using shRNA viruses. Specifically, shRNA‐1 and shRNA‐3 were selected because of their strong inhibitory effects on NR3C1 expression. However, it is better to use sgRNAs (CRISPR tools) that can completely deplete NR3C1 expression. In the future, NR3C1 sgRNAs will be used for the further exploration of NR3C1 function in GC.

## 4 Conclusions

**Figure 8:**
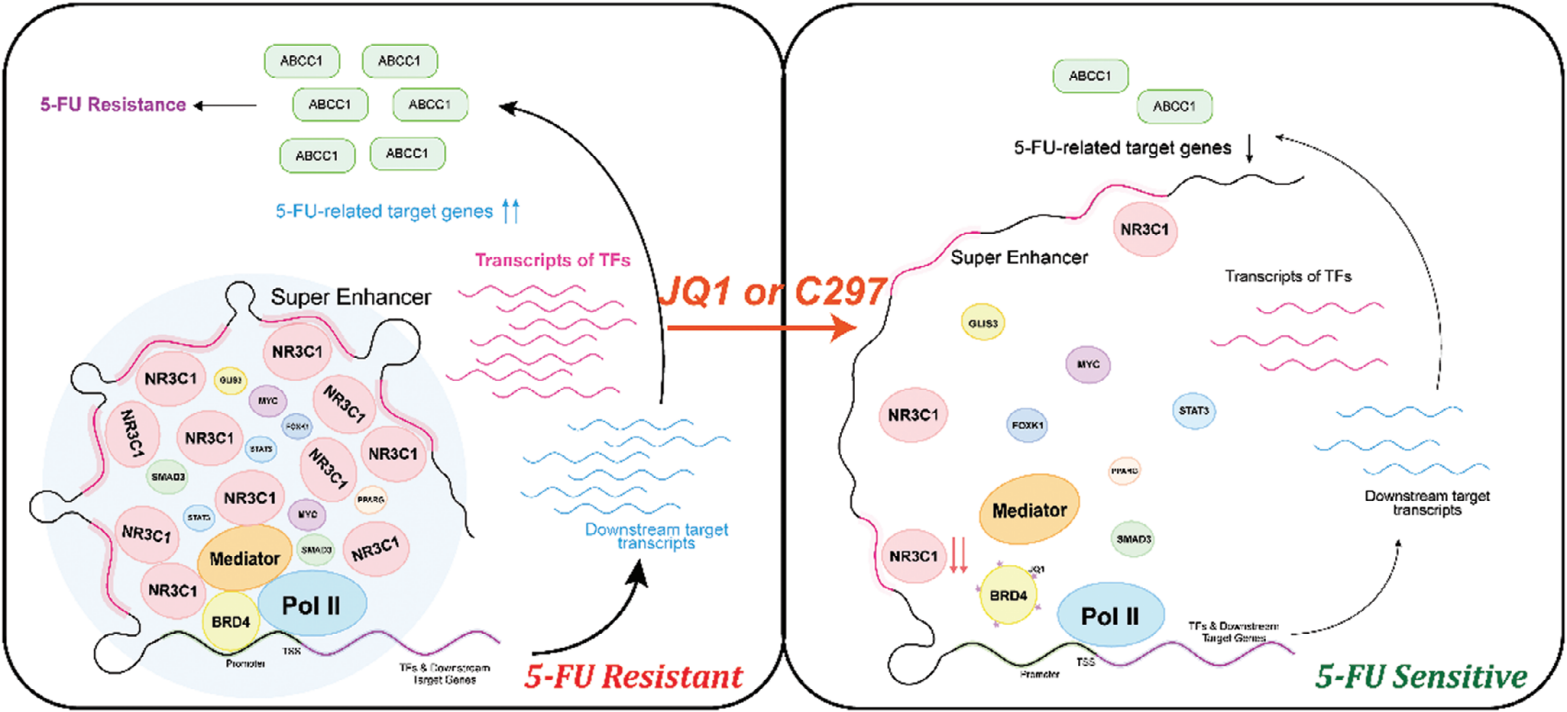
SE‐driven NR3C1 formed phase separation promoting 5‐FU resistance, which was destroyed by JQ1 and Cort108297.

In conclusion, we characterized the SE landscapes of 5‐FU sensitive and resistant GC cells and found that SE‐driven NR3C1 mediated 5‐FU resistance. Together with BRD4 and MED1, NR3C1 formed phase separation in the nucleus and promoted the transcription of downstream genes, leading to 5‐FU resistance. NR3C1 knockdown significantly changed cellular SE composition and inhibited SE function (**Figure** **8**). The epigenetic reader inhibitor JQ1 and NR3C1 specific inhibitor Cort108297 effectively reversed 5‐FU resistance in GC by destroying SE or inhibiting NR3C1. Our findings provide a promising drug combination scheme for patients with GC treated with 5‐FU‐basic chemotherapy to enhance chemotherapy efficiency.

## 5 Experimental Section

### Antibodies and Reagents

The antibodies against Ki‐67(ab16667), MED1 (ab243893), and GAPDH (ab8245) were purchased from Abcam (Cambridge, UK). Antibody against NR3C1 (12 041), BRD4 (13 440), H3K27Ac (8173), and SimpleChIP Plus Enzymatic Chromatin IP Kit (Magnetic Beads) (9005) were purchased from Cell Signaling Technology (Boston, MA, USA). Antibodies against NR3C1 (sc‐393232) were purchased from Santa Cruz (Dallas, TX, USA). A Cell counting Kit‐8 (CCK‐8, CK04) was obtained from Dojindo Molecular Technologies (Kumamoto, Japan). CellTiter‐Glo Luminescent Cell Viability Assay was obtained from Promega (Madison, WI, USA). JQ1 (T2110) and Cort108297 (T15000) were obtained from TargetMol (Boston, MA, USA).

### Cell Culture

Human gastric cancer cells AGS, HS‐746T, and NCI‐N87 were purchased from the American Type Culture Collection (ATCC, USA), and MGC803, MKN28, MKN45, and HGC27 cells were purchased from Shanghai Institutes for Biological Sciences, Chinese Academy of Sciences. Cells were authenticated by STR profiling and free of mycoplasma contamination. Cells cultured in RPMI‐1640 and DMEM medium (Meilunbio, Dalian, China) containing 10% fetal bovine serum (Gibco, Grand Island, NY, USA), 100 U mL^−1^ of penicillin, and 100 µg mL^−1^ of streptomycin, in a humidity culture incubator at 37 °C with 5% CO_2_.

### Dose‐Effect Curves

Cells were plated into 96‐well plates in advance. Medium with 5‐FU/JQ1/C297 at concentrations of different gradients was added to wells and the medium was replaced every day with fresh medium with drugs. After 4 days of culture, the CCK‐8 kit was used according to the manufacturer's instructions. Drug concentrations were converted to Log10C values, and cell viability was normalized based on OD450 values of cells with 0 mg mL^−1^ drug. After applying GraphPad Prism to conduct a nonlinear regression analysis, the IC_50_ values were calculated.

### ChIP‐Seq

ChIP was performed using 10 × 10^6^ cross–linked cells, and sequencing libraries were prepared as previously described.^[^ ^68^ ^]^ The following antibodies were used for ChIP: rabbit anti‐H3K27Ac (CST). ChIP‐seq libraries were sequenced on Illumina HiSeq. Analysis was performed as previously described with some modifications.^[^ ^68^ ^]^ ChIP‐Seq was performed by Genefund Biotech (Shanghai, China).

The SEs were identified with the rank ordering of super‐enhancers (ROSE) algorithm to obtain binding peaks (call peak) within 12.5 kb^[^ ^17^ ^]^ using ChIP‐Seq data. SRGs and TE‐related genes were calculated by “ROSE_geneMapper”.

### Library Construction and RNA‐Seq

Total RNA was extracted from the tissue using TRIzol Reagent according to the manufacturer's instructions (Invitrogen) and genomic DNA was removed using DNase I (TaKara). Then RNA quality was determined by 5300 Bioanalyser (Agilent) and quantified using the ND‐2000 (NanoDrop Technologies). Only high‐quality RNA samples (OD260/280 = 1.8–2.2, OD260/230 ≥ 2.0, RIN ≥ 6.5, 28S:18S ≥ 1.0, >1 µg) were used to construct the sequencing library. RNA‐seq transcriptome library was prepared using Illumina Stranded mRNA Prep (San Diego, CA, USA), SuperScript double‐stranded cDNA synthesis kit (Invitrogen, CA), and Phusion DNA polymerase (NEB) according to the manufacturer's protocol. After quantified by Qubit 4.0, the paired‐end RNA‐seq sequencing library was sequenced with the NovaSeq 6000 sequencer (Illumina).

RNA purification, reverse transcription, library construction, and sequencing were performed at Shanghai Majorbio Bio‐pharm Biotechnology Co., Ltd. (Shanghai, China). Analysis was performed as previously described with some modifications.^[^ ^69^ ^]^

Differential gene expression analysis was performed using the DESeq2 package in R to compare gene expression. Raw read counts obtained from high‐throughput sequencing were first processed to remove low‐quality reads and align them to the human reference genome (GRCh38). The DESeq2 package was then utilized to construct a count table and perform size factor normalization. Differential expression was assessed using a negative binomial generalized linear model. Genes were considered significantly differentially expressed with a log2 fold change (log2FC) ≥ 1 and a *p*‐value ≤ 0.05.

### Enrichment Analysis of Related Genes

Functional‐enrichment analysis including Kyoto Encyclopedia of Genes and Genomes (KEGG) enrichment and Gene Ontology (GO) were performed at Bonferroni‐corrected *p*‐value ≤ 0.05 by Goatools and KOBAS.^[^ ^70^ ^]^ Gene set enrichment analysis (GSEA) analysis was performed to determine the significantly enriched pathways based on the MSigDB database (c2.cp.kegg.v7.2.symbols.gmt gene sets).

### Lentivirus Vectors and Small Interfering RNAs

For the knockdown of NR3C1, target shRNA sequences were subcloned into PGMLV‐HU6‐CMV‐MCS‐ZsGreen1‐PGK‐Puro vector (Genomeditech, Shanghai, China). The shRNA sequences of NR3C1 and siRNA sequences of MED1, BRD4, and TFs in the CRC are listed in Table S1 (Supporting Information). Human full‐length cDNA of NR3C1 was subcloned into PGMLV‐CMV‐MCS‐EF1‐mScarlet‐T2A‐Blasticidin lentivirus vector (Genomeditech). The transfection process and cell selection were performed as previously described.^[^ ^69^ ^]^

### Western Blotting, RNA Extraction, and Quantitative Real‐Time PCR (qRT‐PCR)

Western blotting, RNA extraction, and qRT‐PCR were performed as previously described.^[^ ^69^ ^]^ The sequences of the primers are listed in Table S2 (Supporting Information). The transcription level was normalized to the internal control and determined by a 2^−ΔΔCT^ method.

### Establishment and Culture of Patient‐Derived Organoids (PDO) from GC Patients

PDO establishment was performed as previously described.^[^ ^69^ ^]^ The tumor tissues from GC patients were digested with shaking and filtered through a 70 µm cell strainer (BD Biosciences). After centrifuging, Matrigel was added to the pellet and plated in the 24‐well plate. The shNR3C1 lentivirus, NR3C1 overexpressed lentivirus, and control lentivirus were transfected into organoids. Fluorouracil was added and refreshed every day for ten days and CellTiter‐Glo Luminescent Cell Viability Assay was used to evaluate the viability.

### Fluorescence Recovery after Photobleaching (FRAP)

FRAP was performed as previously described with some modifications.^[^ ^15^, ^31^ ^]^ A lentiviral overexpression plasmid for NR3C1 was generated by cloning the full‐length ORF of the human NR3C1 gene (NM_000176.3) into the vector, followed by a 6 amino acid GS linker sequence “GSGSGS” and mCherry. FRAP was performed on the ZEISS LSM880 Airyscan microscope. The bleach spot was centered on a cluster and images were taken at 1 s intervals for 34 s to measure the fluorescence recovery in the cluster. The integrated intensity of the cluster was determined as a function of time, and background intensity was corrected for overall photo‐bleaching based on a reference region within the same cell, and was normalized to pre‐bleach intensity.

### Co‐Immunoprecipitation (Co‐IP) and Immunoprecipitation‐mass spectrometry (IP‐MS)

Immunoprecipitation was performed using Pierce Co‐Immunoprecipitation Kit (88804, Thermo Fisher) according to the manufacturer's protocol. Antibody against MED1, BRD4, and NR3C1 (10 µg) was covalently cross‐linked to magnetic protein A/G beads as the immunoprecipitation antibody. Lastly, the antigen was eluted and subjected to protein blot analysis with antibodies against MED1, BRD4, and NR3C1. IP‐MS was performed as previously described.^[^ ^69^ ^]^ Protein‐Chip Gold Array (Bio‐Rad) with a Bio‐Rad Protein Chip System Series 4000 mass spectrometer was used for analysis.

### CUT&Tag‐Seq

CUT&Tag assay was performed as described previously with modifications.^[^ ^71^ ^]^ Briefly, 10^5^ cells were washed twice gently with wash buffer. 10 µL Concanavalin A coated magnetic beads (Bangs Laboratories) were added per sample and incubated at RT for 10 min. Remove unbound supernatant and resuspended bead‐bound cells with dig wash buffer and a 1:50 dilution of primary antibody or IgG control antibody (normal mouse IgG: Millipore cat.no.12‐371) incubated on a rotating platform overnight at 4 °C. The primary antibody was removed using a magnet stand. The secondary antibody (Rabbit Anti‐Mouse IgG H&L: Abcam, ab6709) was diluted 1:100 in dig wash buffer and cells were incubated for 60 min. Cells were washed using the magnet stand 2–3 times in dig wash buffer. A 1:100 dilution of pA‐Tn5 adapter complex was prepared in dig‐med buffer (0.01% Digitonin; 20 mm HEPES pH 7.5; 300 mm NaCl; 0.5 mm Spermidine; 1 × Protease inhibitor cocktail) and incubated with cells for 1 h. Cells were washed 2–3 × for 5 min in 1 mL Dig‐med buffer. Then cells were resuspended in a tagmentation buffer (10 mm MgCl2 in Dig‐med Buffer) and incubated at 37 °C for 1 h. DNA was purified using phenol‐chloroform‐isoamyl alcohol extraction and ethanol precipitation.

To amplify libraries, 21 µL DNA was mixed with 2 µL of a universal i5 and a uniquely barcoded i7 primer. A volume of 25 µL NEBNext HiFi 2 × PCR Master mix was added and mixed. The sample was placed in a Thermocycler with a heated lid using the following cycling conditions: 72 °C for 5 min (gap filling); 98 °C for 30 s; 14 cycles of 98 °C for 10 s and 63 °C for 30 s; final extension at 72 °C for 1 min and hold at 8 °C. Library clean‐up was performed with XP beads (Beckman Counter).

### Combination Index

Combination index experiments were performed as described previously.^[^ ^68^ ^]^ JQ1 and 5‐FU were added with serial two‐fold dilutions. CompuSyn was used to generate the combination index values.

### Patient‐Derived Xenograft (PDX) Tumor Models

BALB/c male nude mice (4–6 weeks old, purchased from SPF (Beijing) Biotechnology Co., Ltd., Beijing, China) were housed in specific pathogen‐free cages and used to construct xenograft models. Animal experiments were performed in accordance with the institution's guidelines and animal research principles, and daily care has been provided. After acclimatization for 3 days in an approved facility (water and food at libitum and controlled environmental conditions), experiments were conducted.

PDXs were established with tumors of patients with GC from Ruijin Hospital as described previously.^[^ ^69^ ^]^ For PDX transplantation, tumor fragments of 1–2 mm were subcutaneously engrafted into nude mice under anesthesia. When tumors reached over100 mm^3^, the mice of two cases were separated into groups for treatment with 5‐FU (20 mg kg^−1^, intraperitoneally), and 5‐FU+JQ1 (50 mg kg^−1^, intraperitoneally). Dexamethasone (0.1 mg kg^−1^, intraperitoneally) or C297 (20 mg kg^−1^, intraperitoneally) was given 1 h before chemotherapy administration. Treatment was administered 5 days consecutively per week. Tumor volumes were measured using calipers every 4 days and calculated with the formula: volume (mm^3^) = length × width × width/2. If volumes were over 4000 mm^3^ or the diameter in either dimension was greater than 20 mm (humane endpoint), mice were euthanized. Otherwise, mice were euthanized under anesthesia after the whole treatment, and tumors were removed and weighed.

### Hematoxylin and Eosin (H&E) Analysis and Immunohistochemistry (IHC)

Tumors collected from mice were fixed with 4% paraformaldehyde. Paraffin‐embedded samples were stained with H&E (Sigma). The stained sections were viewed and photographed under a microscope. IHC assay was performed as previously described according to the manufacturer's protocol (Immunostain SP kit, DakoCytomation, USA). Anti‐NR3C1 antibody (1:100), and anti‐Ki67 antibody (1:3000) were used.

### DNA‐FISH Combined with Immunofluorescence

Immunofluorescence was performed as previously described.^[^ ^68^ ^]^ After incubating the cells with the secondary antibodies, cells were washed, fixed with 4% PFA, and washed again. Cells were incubated in 70% ethanol, 85% ethanol, and then 100% ethanol. A probe hybridization mixture was made by mixing 7 µL of FISH Hybridization Buffer, 1 µL of FISH probes, and 2 µL of water. 5 µL of mixture was added on a slide and a coverslip was placed on top. The coverslip was sealed using rubber cement. Genomic DNA and probes were denatured at 78 °C for 5 min and slides were incubated. The coverslip was removed from the slide and incubated in a pre‐warmed wash buffer. Air dry slides and Hoechst stain nuclei. Images were acquired at the Zeiss LSM880 Airscan confocal microscope with 63 × objective.

DNA FISH probes were custom‐designed and generated by Wuhan Boerfu Biotechnology Co., Ltd. to target NR3C1 super‐enhancers. Design Input Region – hg38 chr5:143275273‐143391380.

### Specimens and Ethics

The study received approval from the Human Research Ethics Committee of Ruijin Hospital (Approval No. 2017‐6), in adherence to ethical guidelines outlined in the Declaration of Helsinki. Excluding unpaired tumor‐normal samples and samples of incomplete clinical information, specimens of tumor and adjacent non‐tumor tissues from gastric cancer patients with intact clinical information who underwent D2 gastrectomy were collected. Patients who underwent preoperative treatment were excluded from our study. Animal experiments were conducted in compliance with animal use guidelines and approved by the Laboratory Animal Ethics Committee of Ruijin Hospital (Approval No. SYXK2023‐0038).

### Statistical Analysis

All statistical calculations were performed using GraphPad Prism (version 8.0). All results were presented as the mean ± standard deviation (SD) from triplicate experiments. The sample size for each statistical analysis was indicated in the figure legends. Student's *t*‐tests (two‐tailed) were applied to assess the statistical significance for gene expression (qPCR and WB), ChIP‐qPCR, dual luciferase assays, droplet formation in tubes, and tumor weight. The Spearman correlation test was performed to calculate correlation efficient of mRNA level and IC_50_ values. Cox regression analysis was performed for NR3C1 survival analysis. Comparisons among multiple groups were analyzed by ANOVA. Statistical tests for RNA‐Seq were conducted using R (version 4.2.1), and the DESeq2 package (version 1.38.3) was employed for the differential expression analysis. Differences with a *p*‐value < 0.05 were considered statistically significant.

### Ethics Approval Statement

This study was approved by the Human Research Ethics Committee of Ruijin Hospital (Approval No. 2017‐6), and adhered to the ethical guidelines outlined in the Declaration of Helsinki. Animal experiments were conducted in compliance with the animal use guidelines and approved by the Laboratory Animal Ethics Committee of Ruijin Hospital (Approval No. SYXK2023‐0038).

## Conflict of Interest

The authors declare no conflict of interest.

## Author Contributions

The conceptualization was done by B.L. Data duration was handled by J.Y. Formal analysis was conducted by J.Y., M.C., and Q.S. Methodology was developed by H.F., F.L., Z.X., and B.Y. Investigation was carried out by J.Y., M.C., and Q.S. Resources were provided by C.H., C.Y., Z‐g.Z., J.X., J.L., L.S., and W.D. Software development was done by M.C., Q.S., Y.C., and W.D. Supervision was provided by B.L. and Y‐Y.L. Validation was performed by F.L. and M.C. The original draft was written by J.Y., and the review and editing were done by B.L., Y.Y.L., and Y.C. All the authors read and approved the final manuscript.

## Supporting information



Supporting Information

Supplemental Movie 1

Supplemental Movie 2

Supporting Information

## Data Availability

The data that support the findings of this study are available from the corresponding author upon reasonable request.
