# Supplementary material for: Super‐enhancer Activates Master Transcription Factor NR3C1 Expression and Promotes 5‐FU Resistance in Gastric Cancer
Source: Adv Sci (Weinh). 2024 Dec 27;12(7):2409050. doi: 10.1002/advs.202409050 (PMC11831572; doi:10.1002/advs.202409050)
Supplement: Supplementary file 1 — Supporting Information [file ADVS-12-2409050-s004.docx]

Supporting Information

**Title: Super-enhancer Activates Master Transcription Factor NR3C1 Expression and Promotes 5-FU Resistance in Gastric Cancer**

Authors: Junxian Yu^1,2†^, Mengdi Chen^1^^†^, Qingqing Sang^1†^, Fangyuan Li^1^, Zhuoqing Xu^1^, Beiqin Yu^1^, Changyu He^1^, Liping Su^1^, Wentao Dai ^3^, Chao Yan^1^, Zheng-gang Zhu^1^, Jiazeng Xia^4^, Jianfang Li^1^, Haoran Feng^1^, Yunqin Chen ^3*^, Yuan-Yuan Li^3*^, Bingya Liu^1^*****

**
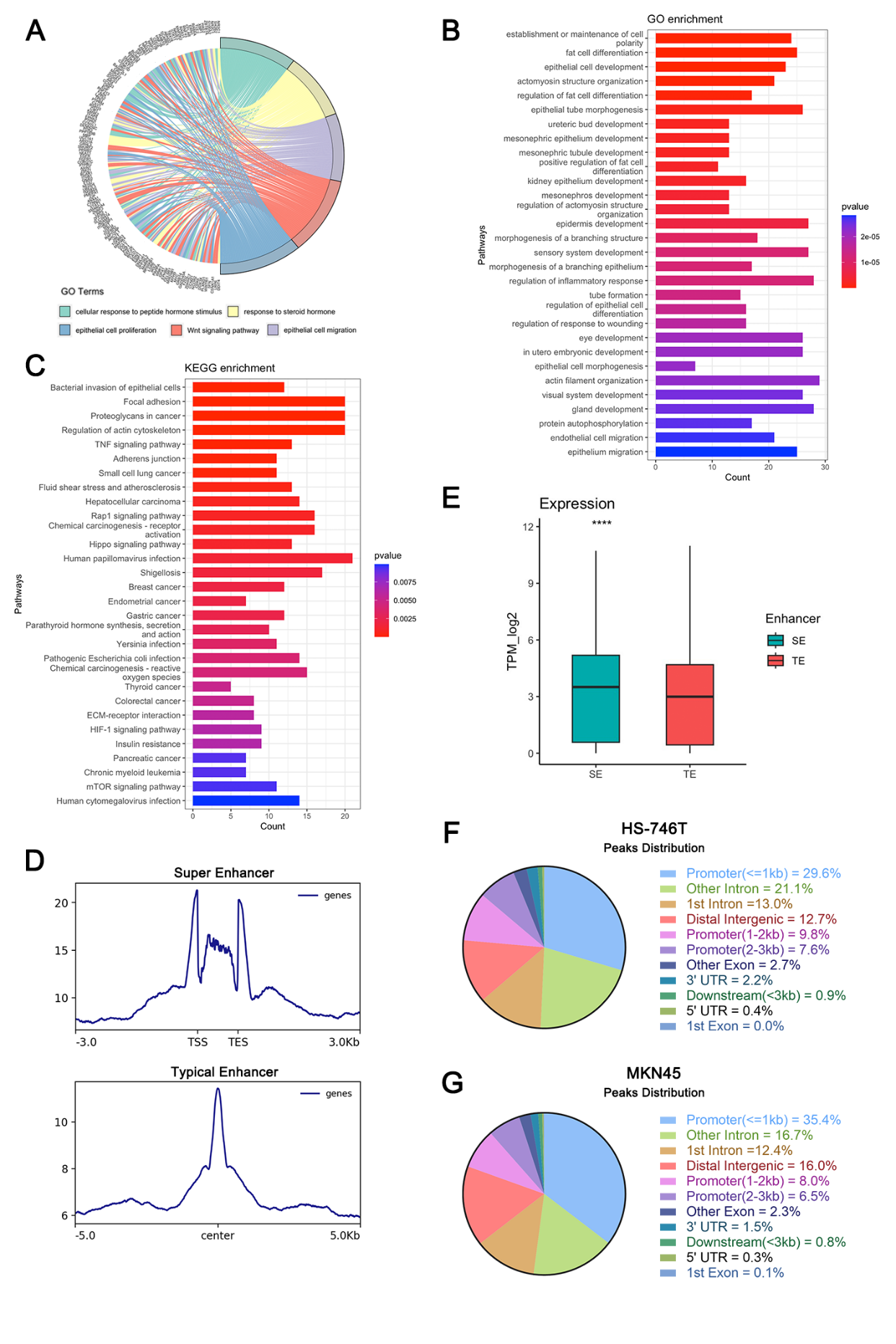
**

**Figure. S1. SE landscape of HS-746T and MKN45**

A. SE-related genes (SRGs) enriched in epithelial related pathways and Wnt related pathways in HS-746T cells. B. GO enrichment analysis of SRGs in MKN45 cells. C. KEGG enrichment analysis of SRGs in MKN45 cells. D. Peak graphs of SEs and TEs in MKN45. E. Transcriptional levels of SRGs and TE-related genes in MKN45 cells (Student's t tests, two-tailed). F. Peak distribution of H3K27Ac ChIP-Seq data in HS-746T cells. G. Peak distribution of H3K27Ac ChIP-Seq data in MKN45 cells.


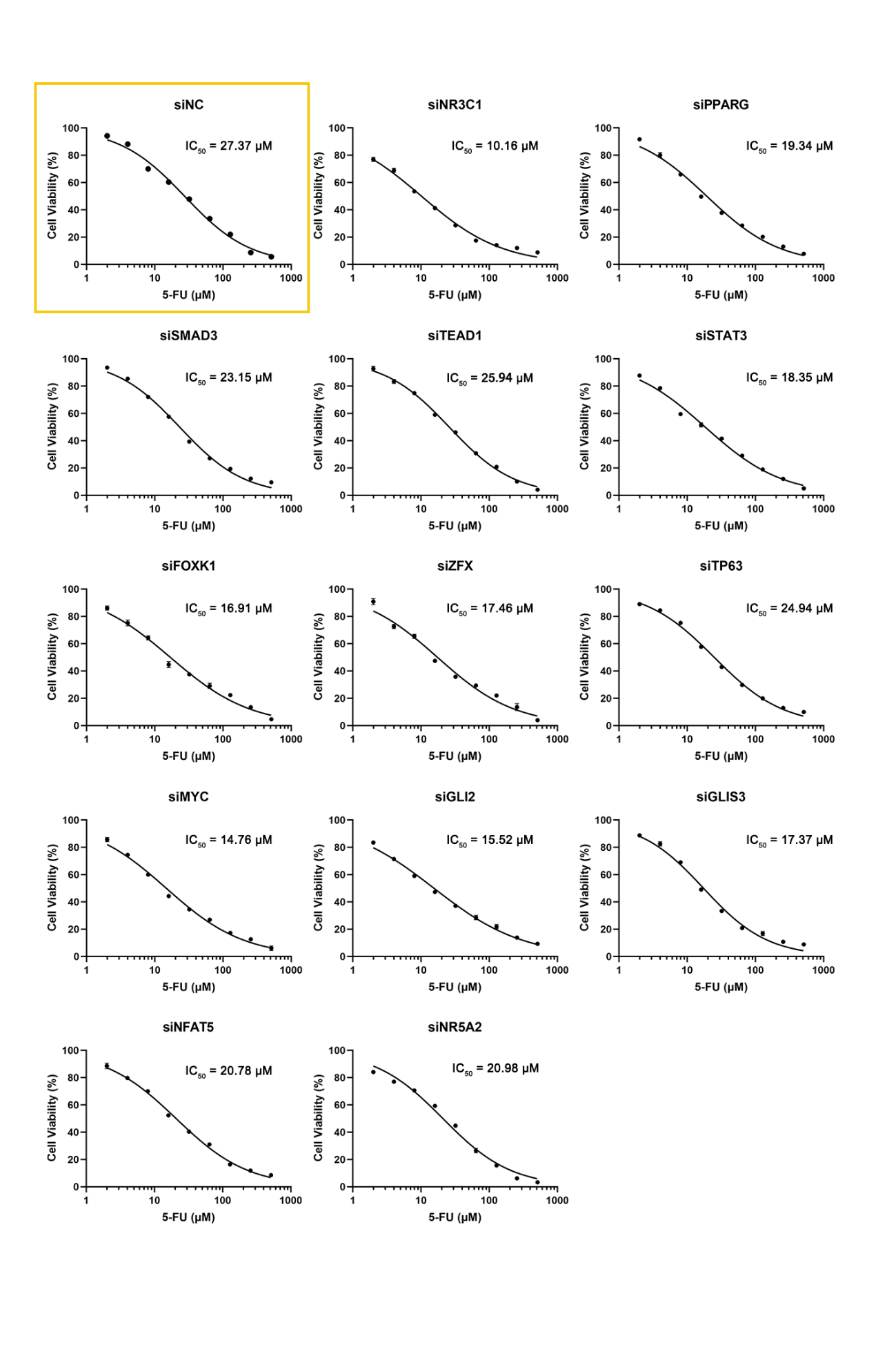


**Figure. S2. Drug curves and IC_50_ values of HS-746T with every siTFs**

**
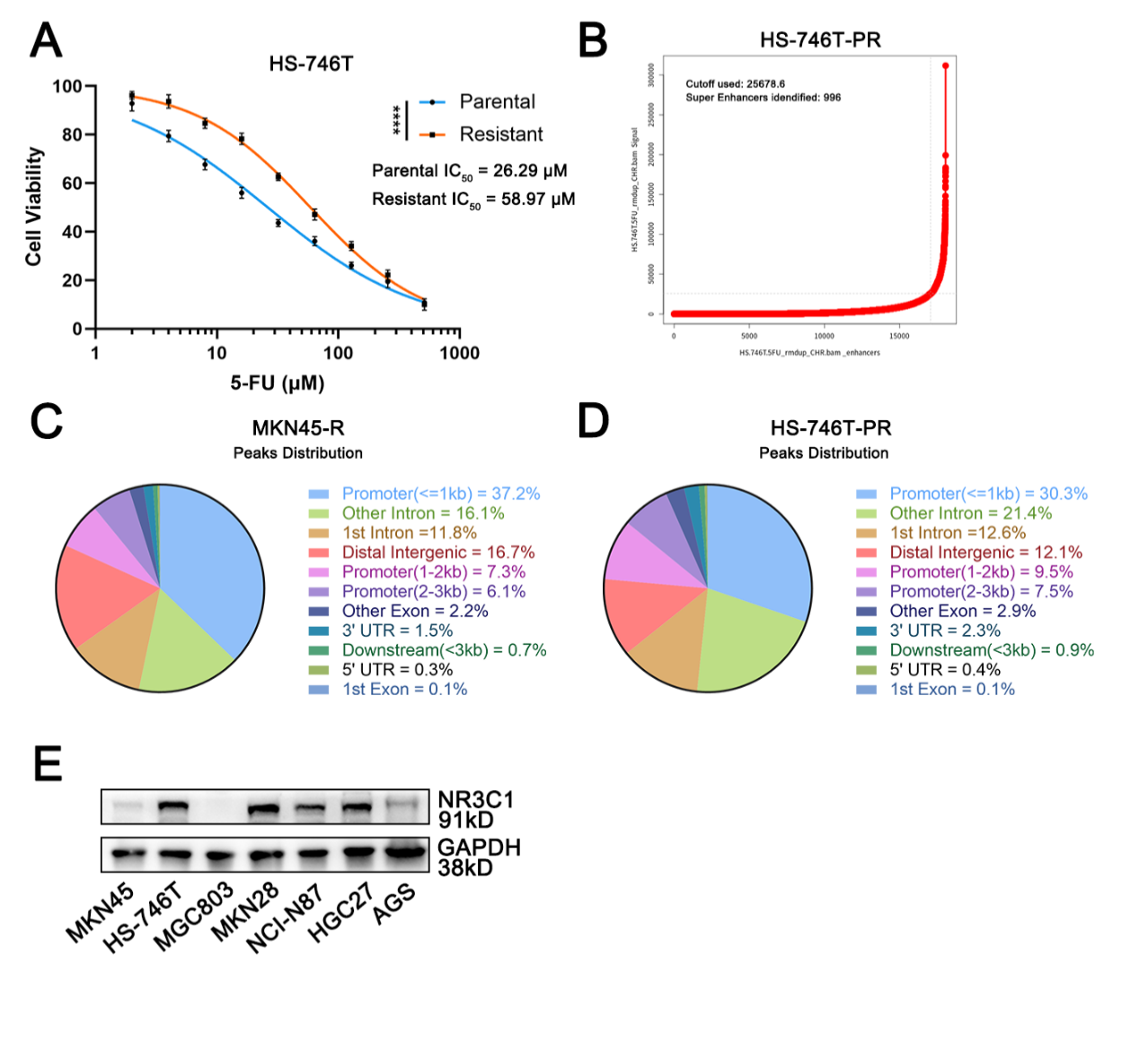
**

**Figure. S3. SE landscape of HS-746T-PR and MKN45-R**

A. IC_50_ values of 5-FU in HS-746T-PR and parental cells (ANOVA, two-tailed). B. SE curve of HS-746T-PR GC cells. C. Distribution of H3K27Ac peaks on chromosomes of MKN45-R cells. D. Distribution of H3K27Ac peaks on chromosomes of HS-746T-PR cells. E. NR3C1 expression in seven GC cell lines.


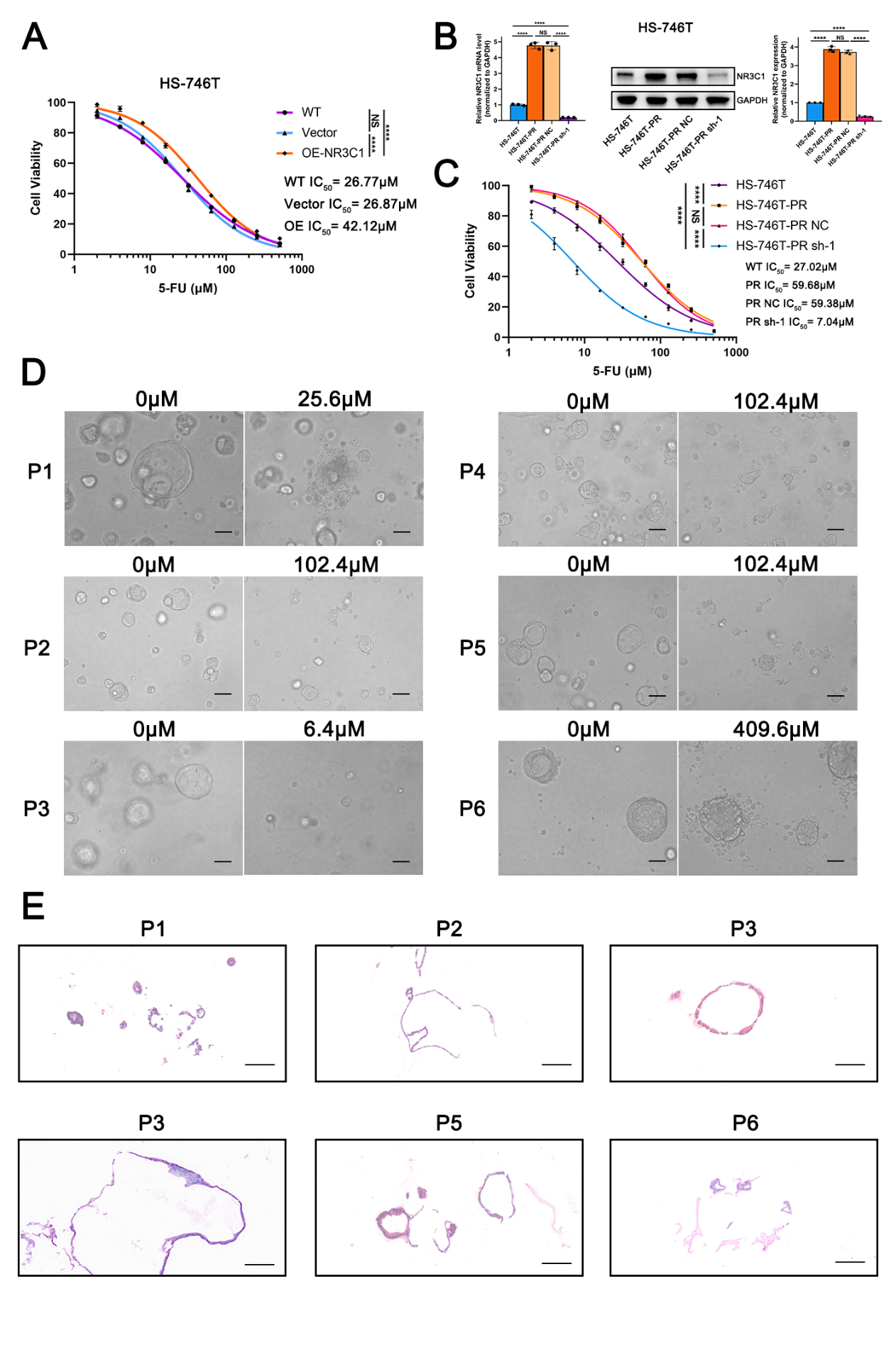


**Figure. S4. High NR3C1 expression promoted the 5-FU resistance of GC cells and organoids**

A. IC_50_ curves of 5-FU in HS-746T-OE cells (ANOVA, two-tailed). B. NR3C1 knockdown detection in mRNA (left) and protein (right) levels in HS-746T-PR cells (ANOVA, two-tailed). C. The 5-FU IC_50_ values of HS-746T, HS-746T-PR, HS-746T-PR NC and HS-746T-PR sh-1 cells (ANOVA, two-tailed). D. Photos around the IC_50_ values of the organoids P1-6. Scale: 100 μm. E. H&E staining of organoids P1-6. Scale: 100 μm. *p<0.05, **p<0.01, ***p<0.001, ****p <0.0001.

**
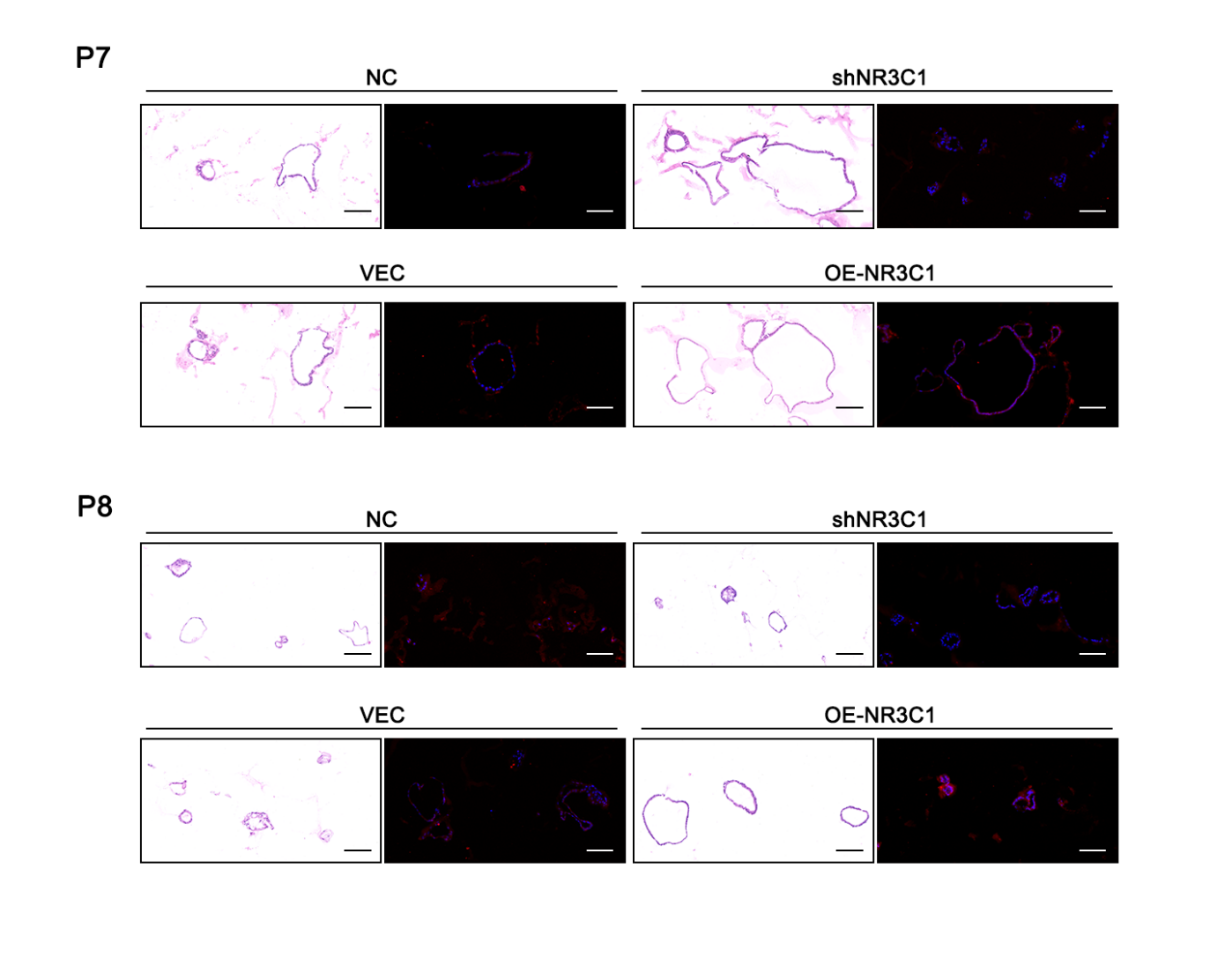
**

**Figure. S5. H&E staining and NR3C1 immunofluorescence of P7-8**

A. H&E staining and NR3C1 immunofluorescence levels of organoids P7-8. Scale: 100 μm. *p<0.05, **p<0.01, ***p<0.001, ****p <0.0001.

**
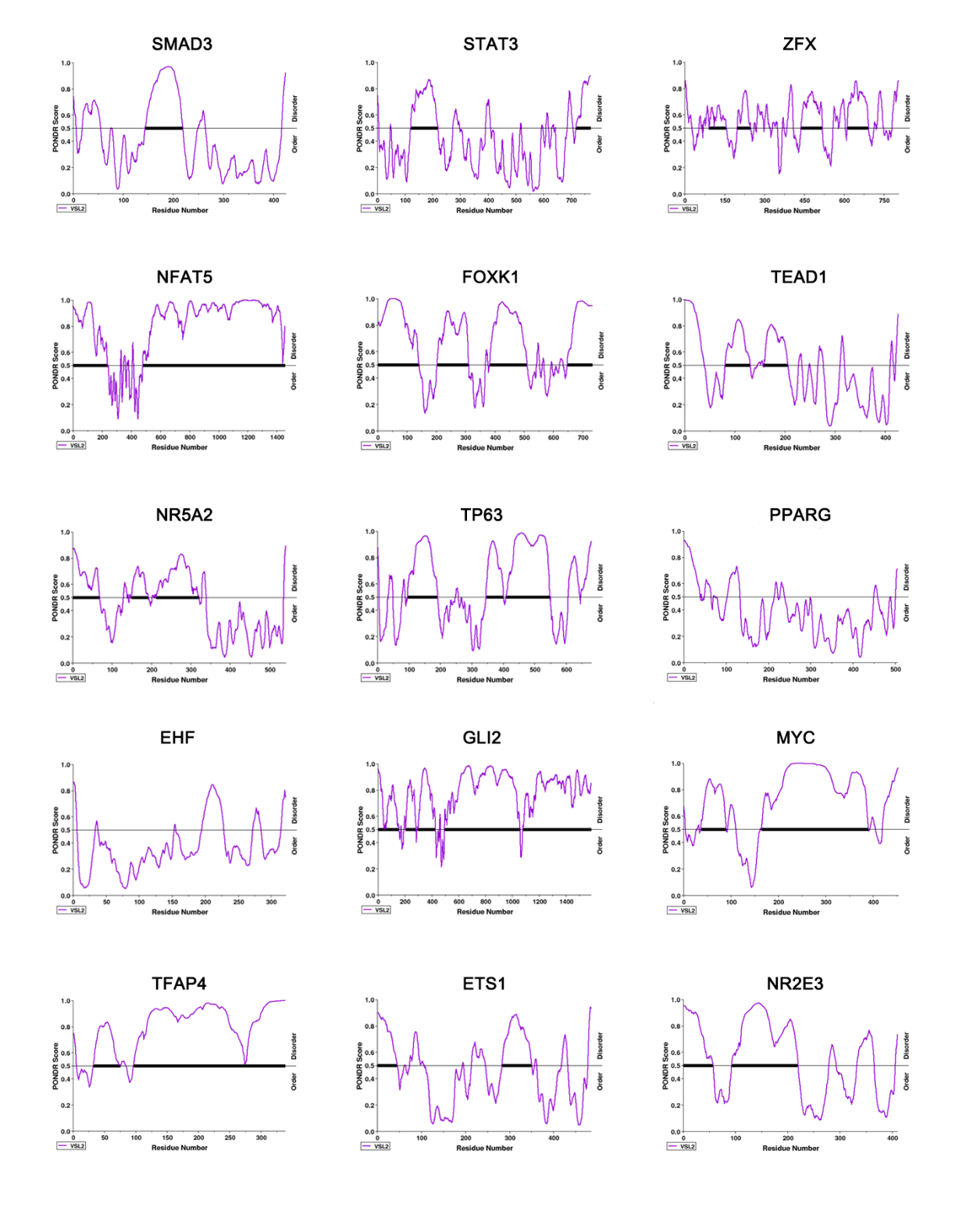
**

**Figure. S6. IDRs of every TF protein in the potential CRC calculated with Ponder**

**
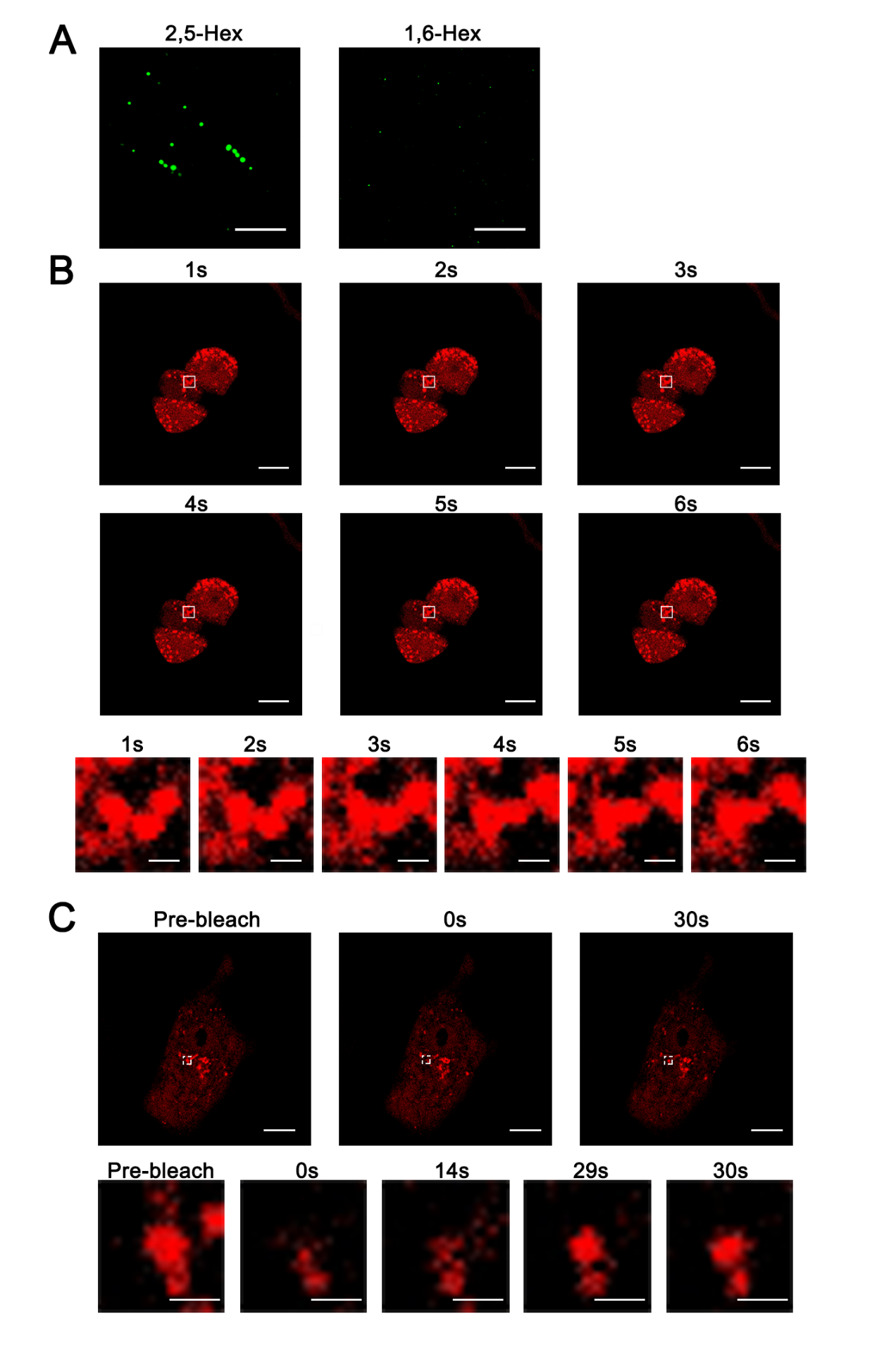
**

**Figure. S7. NR3C1 immunofluorescence and FRAP**

A. Pictures of droplets with the use of 1, 6-hexadiol and 2, 5-hexadiol. Scale: 5 μm. B. Droplets fusion of mCherry-NR3C1 protein in the nucleus of HS-746T. Top: scale: 10 μm, bottom: scale: 1 μm. C. Time series of FRAP images of mCherry-NR3C1 protein in the nucleus of HS-746T. Top: scale: 10 μm, bottom: scale: 1 μm. *p<0.05, **p<0.01, ***p<0.001, ****p <0.0001.

**
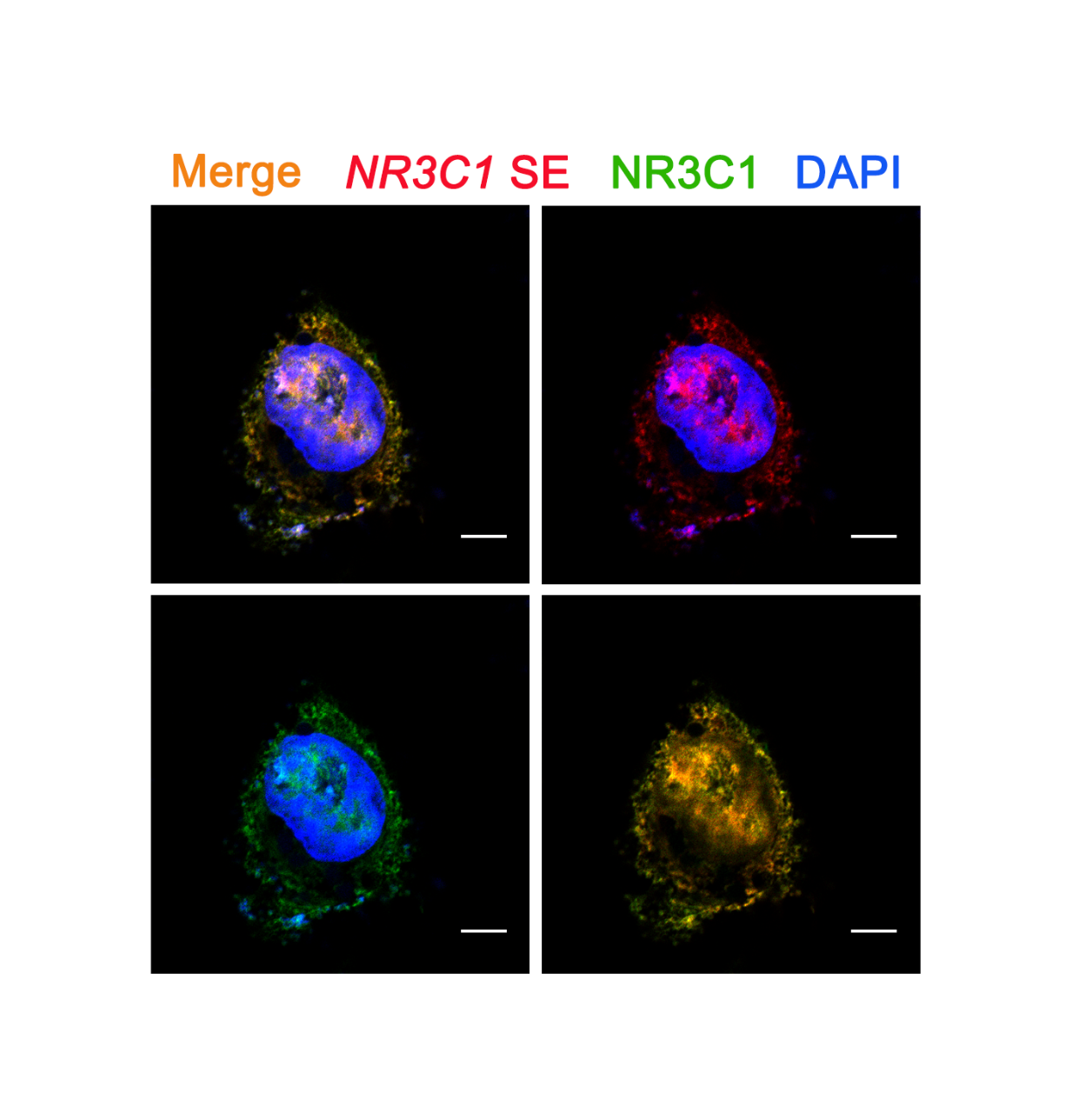
**

**Figure. S8.** **DNA-FISH** **at NR3C1 SE combined with immunofluorescence**


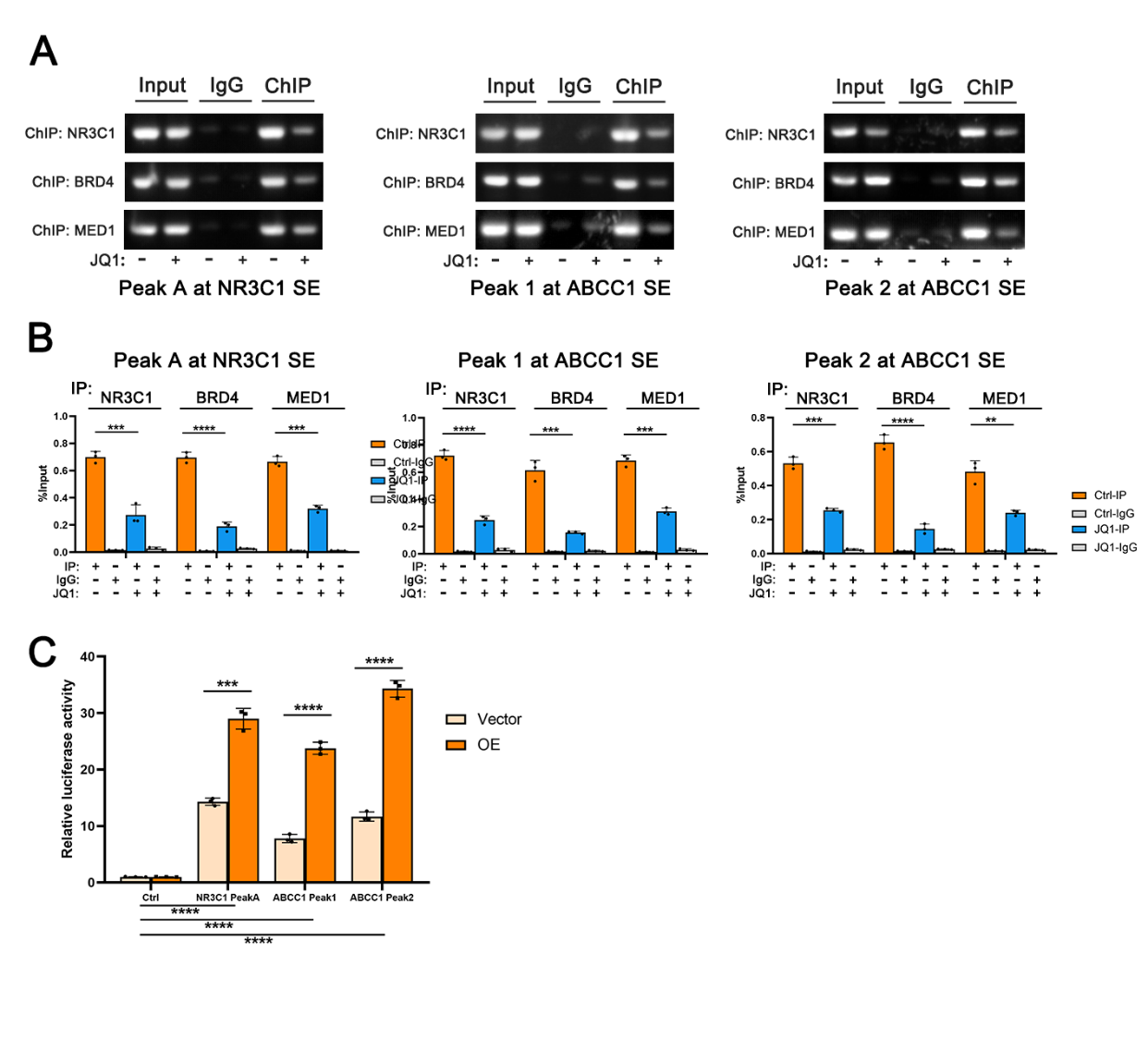


**Figure. S9. NR3C1, BRD4 and MED1 bound to the SEs of NR3C1 and ABCC1.**

A. ChIP-PCR of NR3C1, BRD4 and MED1 bound to the SEs of NR3C1 and ABCC1. B. ChIP-qPCR was performed using antibodies against NR3C1, BRD4, MED1 and IgG (negative control) (Student's t tests, two-tailed). C. Dual luciferase reporter assays in HS-746T vector and HS-746T NR3C1-overexpressed cells (ANOVA, two-tailed).

**
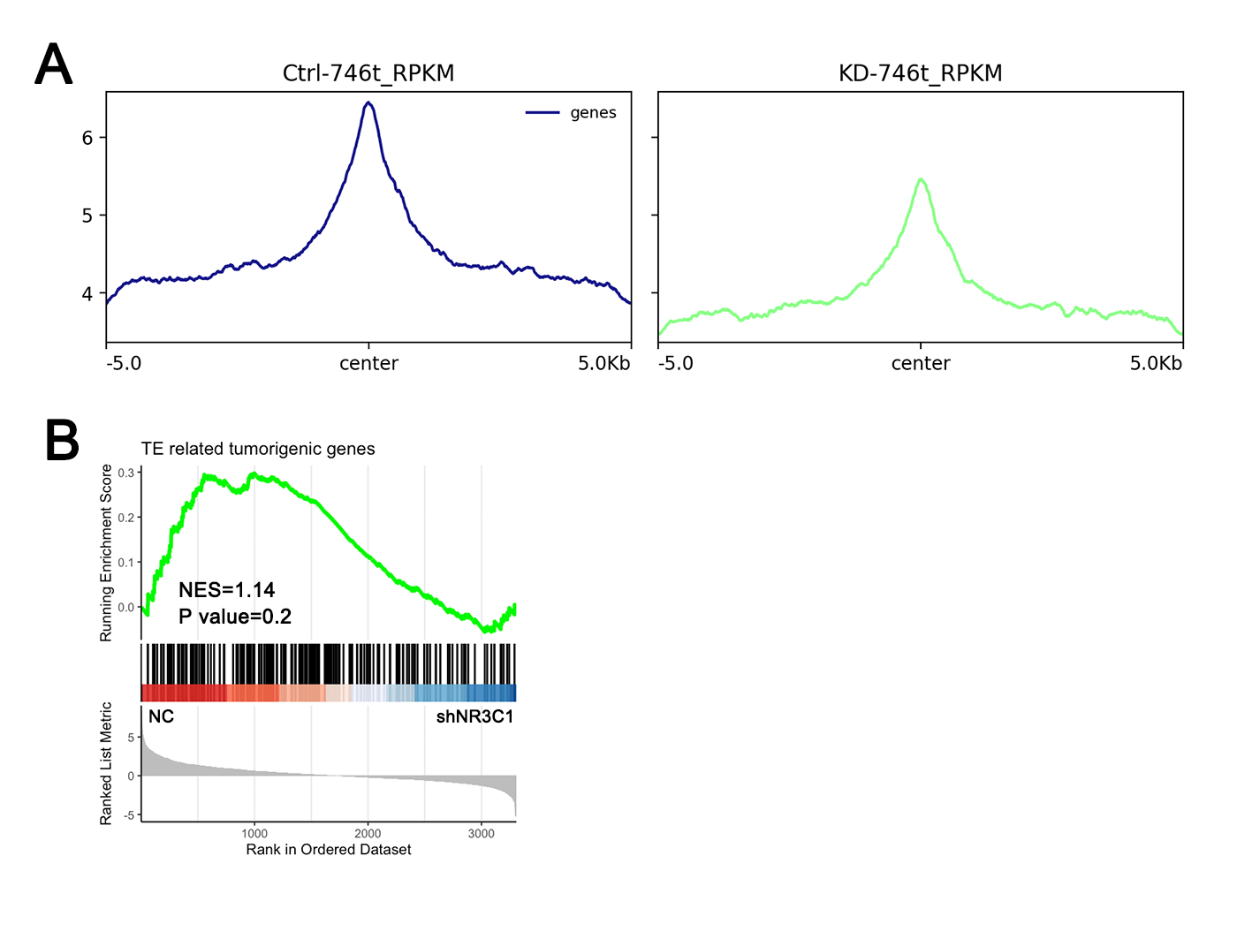
**

**Figure. S10.** A. Peak graphs of TEs in HS-746T knockdown and control cells. B. GSEA of TE-associated gene set in control versus shNR3C1 cells.


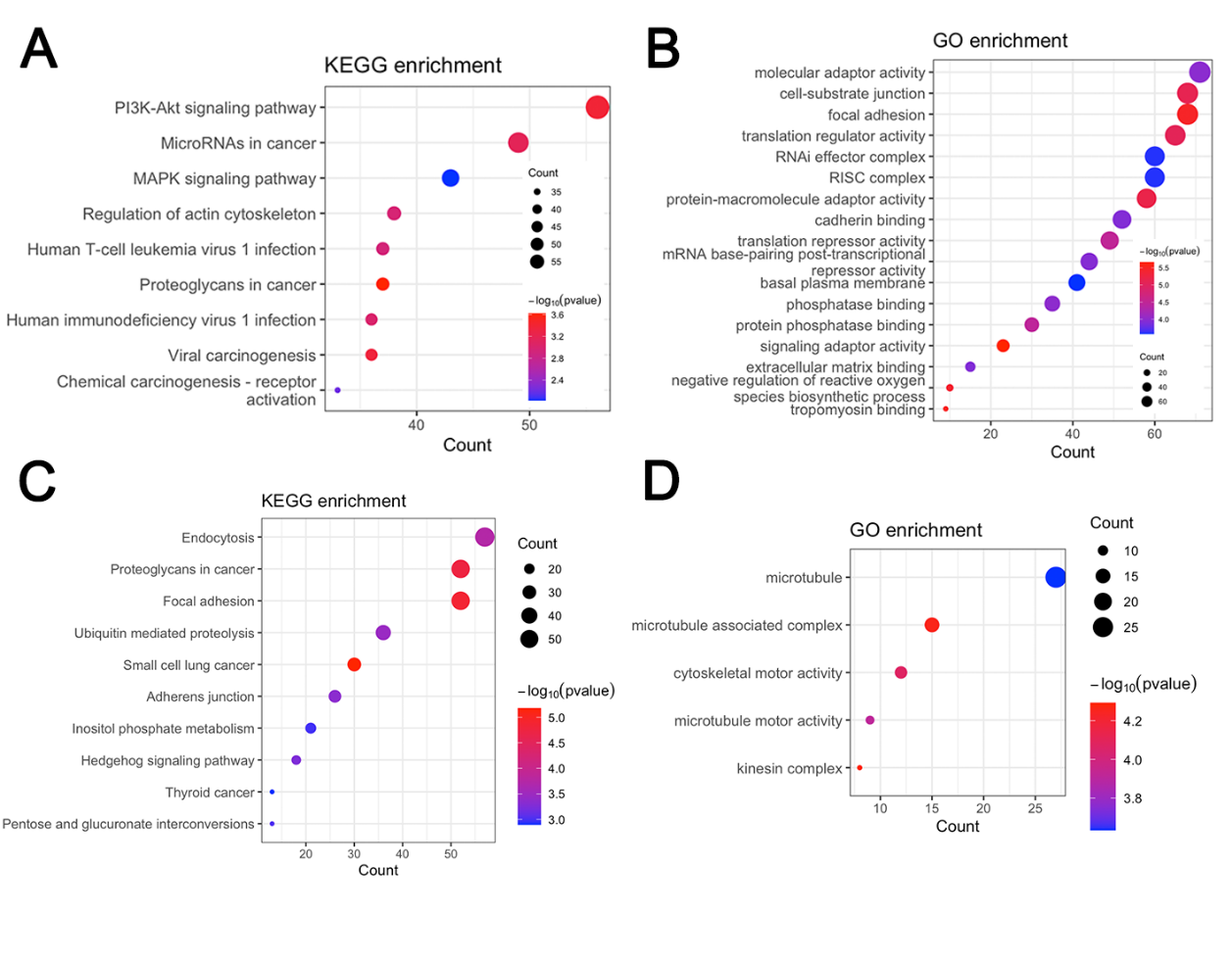


**Figure. S11.** A. KEGG enrichment analysis of SE related genes in HS-746T-PR. B. GO enrichment analysis of SE related genes in HS-746T-PR. C. KEGG enrichment analysis of differentially highly expressed genes in control group. D. GO enrichment analysis of differentially highly expressed genes in knockdown group.

**
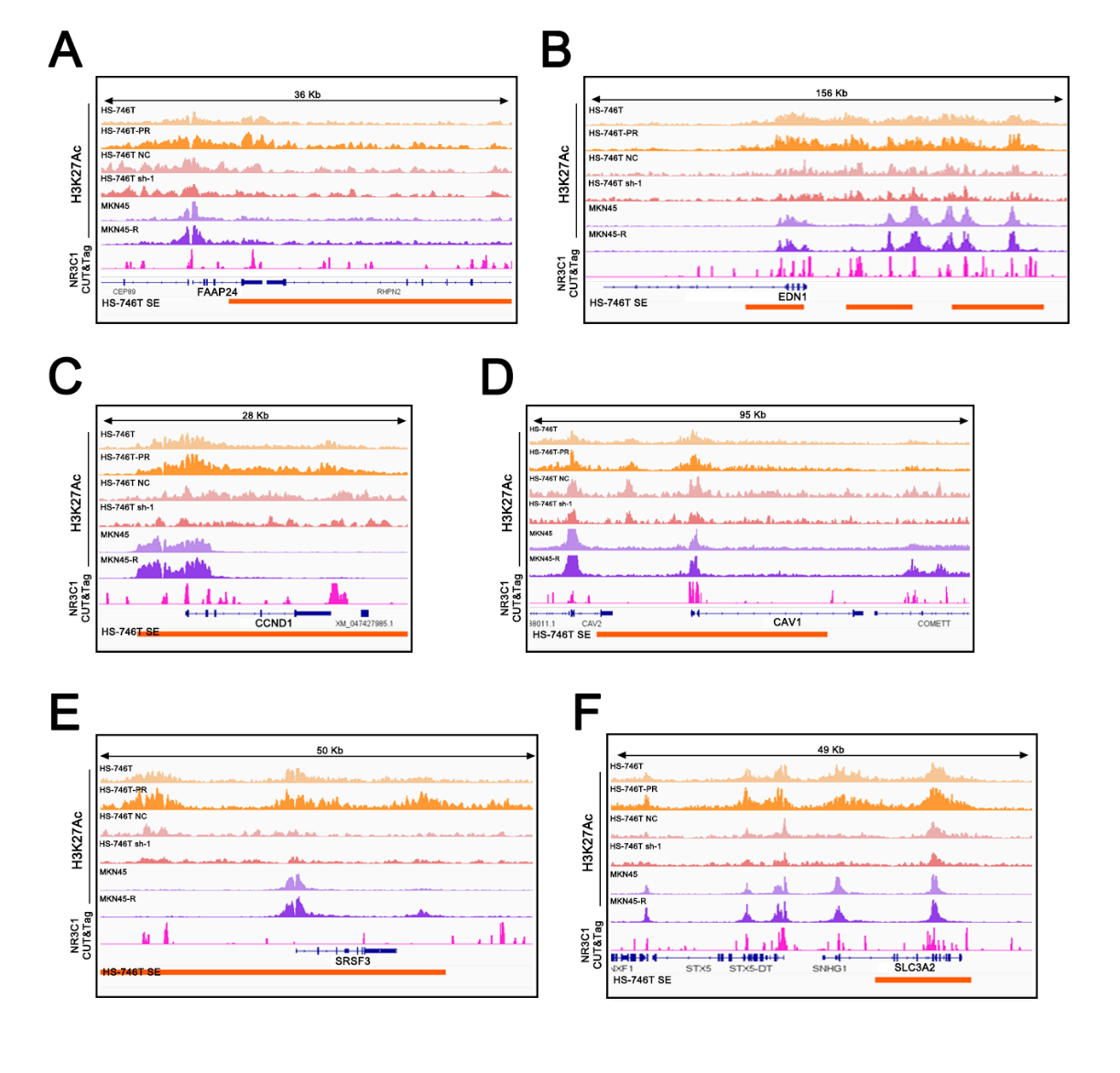
**

**Figure. S12. IGV map of around NR3C1 downstream genes**

A. IGV map of H3K27Ac peak in HS-746T, HS-746T-PR, HS-746T NC and HS-746T-sh-1, MKN45 and MKN45-R groups and NR3C1 CUT&Tag binding peaks around FAAP24. B. IGV map around EDN1 in indicated groups. C. IGV map around CCND1 in indicated groups. D. IGV map around CAV1 in indicated groups. E. IGV map around SRSF3 in indicated groups. F. IGV map around SLC3A2 in indicated groups.

**
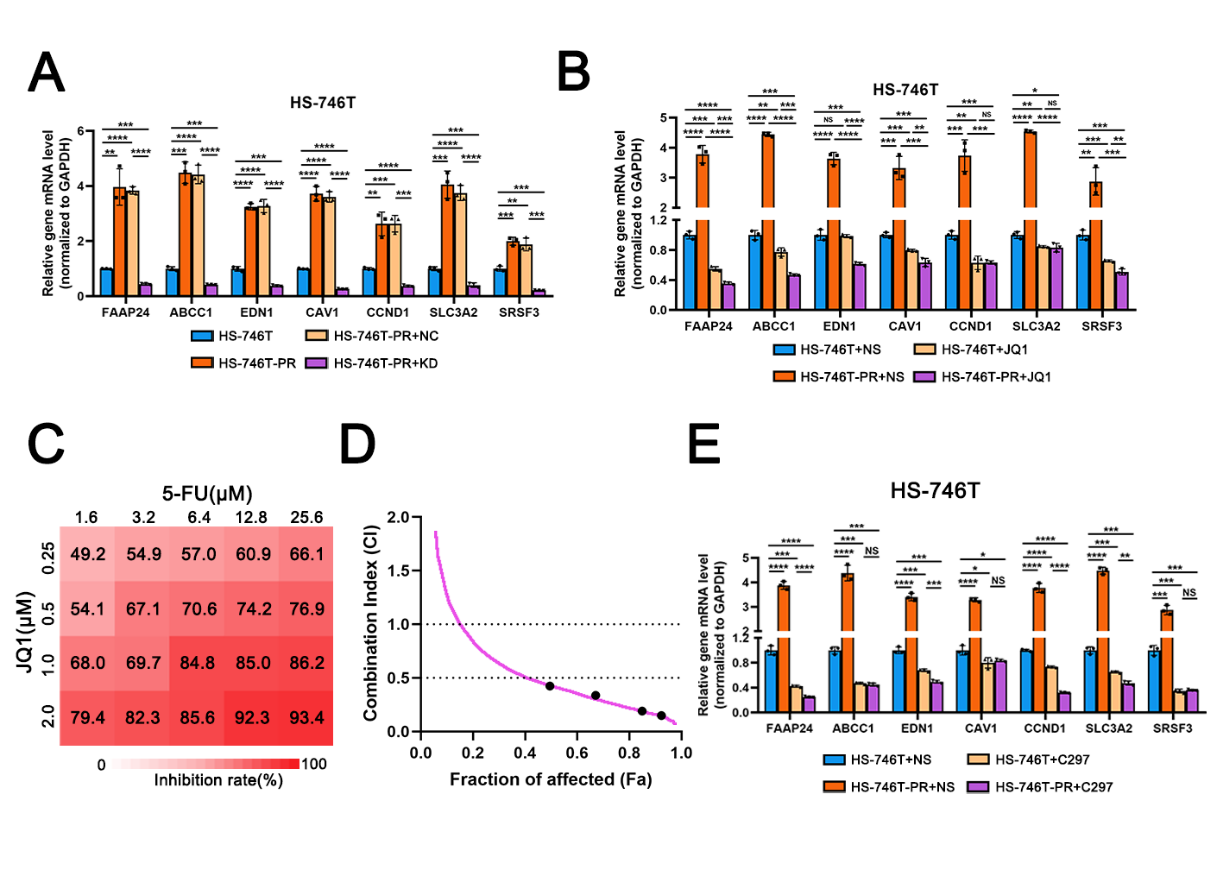
**

**Figure. S13. SE characteristics and regulation of SE-driven NR3C1 downstream genes**

A. The mRNA levels of 5-FU-related gene sets in HS-746T, HS-746T-PR, HS-746T-PR NC, and HS-746T-PR KD groups (ANOVA, two-tailed). B. The mRNA levels of 5-FU-related gene sets in HS-746T+NS, HS-746T-PR+NS, HS-746T+JQ1, HS-746T-PR+JQ1 groups (ANOVA, two-tailed). C. Synergistic effects of the combined use of JQ1 and 5-FU in HS-746T. The color scale for drug inhibition values. D. Fa (Fraction of affected)-CI (Combination index) plots in HS-746T for the combination of JQ1 and 5-FU. CI values of <0.5 indicate moderate synergistic effects. E. The mRNA levels of 5-FU-related gene sets in HS-746T+NS, HS-746T-PR+NS, HS-746T+C297, HS-746T-PR+C297 groups (ANOVA, two-tailed). *p<0.05, **p<0.01, ***p<0.001, ****p <0.0001.


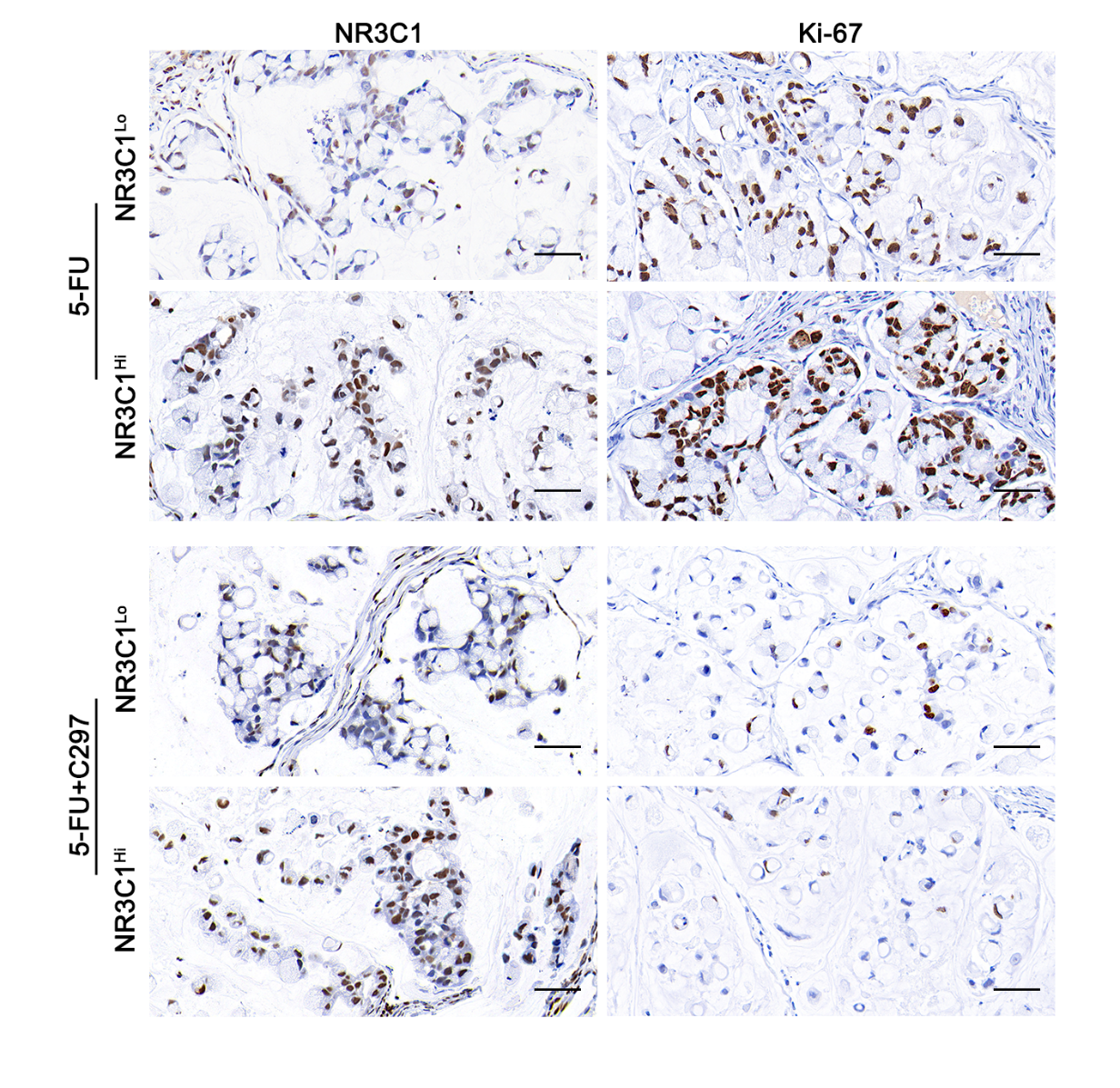


**Figure. S14. IHC staining (KI-67 and NR3C1) of NR3C1^Hi^ and NR3C1^Lo^ PDX tumors in 5-FU and 5-FU+C297 groups.** **Scale bars: 50 μm.**

**Table. S1 shRNA** **sequences and siRNA sequences**

| **Name** | **5’-3’ Sequences** |
| --- | --- |
| *NR3C1*-shRNA-1 | GTGTCACTGTTGGAGGTTATT |
| *NR3C1*-shRNA-2 | CCTGGATGTTTCTTATGGCAT |
| *NR3C1*-shRNA-3 | CACAGGCTTCAGGTATCTTAT |
| *SMAD3* siRNA 5’-3’ | GCCUCAGUGACAGCGCUAUUUTT |
| *STAT3* siRNA 5’-3’ | GCACAAUCUACGAAGAAUCAATT |
| *ZFX* siRNA 5’-3’ | GUCGGAAAUUGAUCCUUGUAATT |
| *NFAT5* siRNA 5’-3’ | GCUGGUGCUUUGAAUGUAATT |
| *NR5A2* siRNA 5’-3’ | GCGUUGUCCUUACUGUCGUUUTT |
| *PPARG* siRNA 5’-3’ | CAGCAUUUCUACUCCACAUUATT |
| *MED1* siRNA 5’-3’ | GCCGAGUUCCUCUUAUCCUAATT |
| *BRD4* siRNA 5’-3’ | CCUGGAGAUGACAUAGUCUUATT |
| *TEAD1* siRNA 5’-3’ | CCGAUUUGUAUACCGAAUAAATT |
| *MYC* siRNA 5’-3’ | CCUGAGACAGAUCAGCAACAATT |
| *GLIS3* siRNA 5’-3’ | CGGGCAUUACAGUGUAUGAUUTT |
| *GLI2* siRNA 5’-3’ | GUUCCUGAACAUGAUGACCUATT |
| *TP63* siRNA 5’-3’ | GGACAGCAGCAUUGAUCAATT |

**Table. S2 Primers**

| **Name** | **5’-3’ Sequences** |
| --- | --- |
| *NR3C1*-human-F | ACAGCATCCCTTTCTCAACAG |
| *NR3C1*-human-R | AGATCCTTGGCACCTATTCCAAT |
| *GAPDH*-human-F | GGAGCGAGATCCCTCCAAAAT |
| *GAPDH*-human-R | GGCTGTTGTCATACTTCTCATGG |
| *ABCC1*-human-F | GTCGGGGCATATTCCTGGC |
| *ABCC1*-human-R | CTGAAGACTGAACTCCCTTCCT |
| *CAV1*-human-F | GCGACCCTAAACACCTCAAC |
| *CAV1*-human-R | ATGCCGTCAAAACTGTGTGTC |
| *CCND1*-human-F | GCTGCGAAGTGGAAACCATC |
| *CCND1*-human-R | CCTCCTTCTGCACACATTTGAA |
| *EDN1*-human-F | AGAGTGTGTCTACTTCTGCCA |
| *EDN1*-human-R | CTTCCAAGTCCATACGGAACAA |
| *EHF*-human-F | GCTCAGCTATGGGGTAAAAAGAA |
| *EHF*-human-R | ATCCACACGCTCCAGAATTTC |
| *FAAP24*-human-F | TTAAGCTCATTTTCGAGGATGGC |
| *FAAP24*-human-R | CCCGAACAAGCCTCTTTCTGT |
| *FOXK1*-human-F | CAGTTACCGCTTTGTGCAGAA |
| *FOXK1*-human-R | CGGCTTTGACTCATCCTTGG |
| *GLI2*-human-F | CCCCTACCGATTGACATGCG |
| *GLI2*-human-R | GAAAGCCGGATCAAGGAGATG |
| *GLIS3*-human-F | GTTCAGCGACTGGGACTCATT |
| *GLIS3*-human-R | CCCTCTGTAAGCTAGGACTGAT |
| *MYC*-human-F | GTCAAGAGGCGAACACACAAC |
| *MYC*-human-R | TTGGACGGACAGGATGTATGC |
| *NFAT5*-human-F | GCTGGATAACAGTCGGATGTC |
| *NFAT5*-human-R | GCCTCTGCTTTGGATTTCGTT |
| *NNMT*-human-F | ATATTCTGCCTAGACGGTGTGA |
| *NNMT*-human-R | TCAGTGACGACGATCTCCTTAAA |
| *NR5A2*-human-F | CTTTGTCCCGTGTGTGGAGAT |
| *NR5A2*-human-R | GTCGGCCCTTACAGCTTCTA |
| *PPARG*-human-F | GGGATCAGCTCCGTGGATCT |
| *PPARG*-human-R | TGCACTTTGGTACTCTTGAAGTT |
| *SLC3A2*-human-F | TGAATGAGTTAGAGCCCGAGA |
| *SLC3A2*-human-R | GTCTTCCGCCACCTTGATCTT |
| *SMAD3*-human-F | TGGACGCAGGTTCTCCAAAC |
| *SMAD3*-human-R | CCGGCTCGCAGTAGGTAAC |
| *SRSF3*-human-F | TGGCAACAAGACGGAATTGGA |
| *SRSF3*-human-R | CAAAGCCGGGTGGGTTTCTA |
| *STAT3*-human-F | CAGCAGCTTGACACACGGTA |
| *STAT3*-human-R | AAACACCAAAGTGGCATGTGA |
| *TEAD1*-human-F | ATGGAAAGGATGAGTGACTCTGC |
| *TEAD1*-human-R | TCCCACATGGTGGATAGATAGC |
| *TYMS*-human-R | CTGCTGACAACCAAACGTGTG |
| *TYMS*-human-R | GCATCCCAGATTTTCACTCCCTT |
| *TP63*-human-F | CCACCTGGACGTATTCCACTG |
| *TP63*-human-R | TCGAATCAAATGACTAGGAGGGG |
| *ZFX*-human-F | TTGCTGAAATCGCTGACGAAG |
| *ZFX*-human-R | GCAATCGGCATGAAGGTTTTGAT |
| *NR3C1* SE Peak A-human-F | GGACAGATTGGTGGGGGAAG |
| *NR3C1* SE Peak A-human-R | TGCTTGAGAAGCTCACCTGG |
| *ABCC1* SE Peak 1-human-F | CGGGACAACCTGTTTGCTTG |
| *ABCC1* SE Peak 1-human-R | AGTGACTCAGCACGAAGTGG |
| *ABCC1* SE Peak 2-human-F | CAAGTTGATGGGCAAAGGGC |
| *ABCC1* SE Peak 2-human-R | GTGCACCCAGCTGTTATCCT |
